# Sequence-encoded determinants of regional mutational plasticity: comparative analysis of PE_PGRS genes in *Mycobacterium tuberculosis* and other bacteria

**DOI:** 10.1038/s41598-026-47170-w

**Published:** 2026-05-02

**Authors:** Veranika Slizen, Henadz Hurevich

**Affiliations:** Department of Tuberculosis Diagnosis and Treatment, Republican Scientific and Practical Center for Pulmonology and Phthisiatry, Dolginovsky Trakt, 157, 220080 Minsk, Belarus

**Keywords:** Computational biology and bioinformatics, Evolution, Genetics, Microbiology

## Abstract

The PE_PGRS gene family in *Mycobacterium tuberculosis* exhibits extensive sequence variability across genotypes, which is consistent with antigenic divergence. Here we investigate how *Mtb*—despite lacking horizontal gene transfer—balances genomic stability with adaptive plasticity. Comparative analysis of 88 bacterial genomes reveals that PE_PGRS genes exhibit features facilitating mutability, including a significantly elevated CGGC tetramer density (mean 4.97 per 100 nt; range 1.7–7.4) compared with the genome-wide average (1.62 per 100 nt; *p* = 0.011) and depleted in out-of-frame stop codons, potentially conferring robustness to 1-nt and 2-nt frameshifts. Computational predictions suggest that CGGC motifs may promote secondary DNA structures, potentially destabilizing replication and contributing to replication errors, while the scarcity of out-of-frame stop codons allows continued translation beyond frameshifts, leading to changes in protein sequence and length. This dual organization may contribute to the observed adaptability of *M. tuberculosis* and could highlight a broader principle by which some pathogens evolve under strong constraints on horizontal gene transfer. We propose that CGGC-rich regions may function as programmed mutational hotspots across a wide range of microorganisms.

**Supplementary Information:**

The online version contains supplementary material available at 10.1038/s41598-026-47170-w.

## Introduction

*Mycobacterium tuberculosis* (*Mtb*) remains one of the most successful human pathogens^1–5^. The incidence of tuberculosis (TB) persists despite long-standing national and international programs to control the spread of TB^6–10^. *Mtb* has successfully overcome the selective pressure exerted by the use of an ever-expanding array of anti-TB drugs^10–16^. Unlike many other bacteria, *Mtb* lacks horizontal gene transfer mechanisms^17–19^. *Mtb* were conceptualized as a group of genes moving together through time and space^20^. To actualize high adaptive fitness, *Mtb* may maintain mechanisms with promutagenic potential that provide mutational variation at loci important for adaptation and survival of *Mtb* in equilibrium changing external factors^21^. Understanding how genomes simultaneously maintain stability and permit adaptive variability is a central question in evolutionary biology.

Approximately 10% of the coding capacity of the *Mtb* genome is associated with the PE (including PE_PGRS) and PPE gene family, present only in the genus Mycobacterium^22^. Many researchers confirm the considerable genetic variation of PE_PGRS genes basing on the presence of multiple single nucleotides polymorphisms, deletions, and insertions ^23–32^. This variability stands in contrast to the relatively conserved nature of the core genome, suggesting the presence of endogenous mechanisms behind such site-specific changes. There is a need for studies that bridge evolutionary, structural, and immunological gaps to unravel the contribution of PE/PPE proteins to the adaptive success of *Mtb*^33^*.*

Currently, spontaneous mutations are commonly defined as replication errors, which presumes uniform occurrence of mutations throughout the genome. However, this assumption neglects the existence of two opposites – highly conserved and hypervariable regions that enable rapid adaptation, as well does not reflect the long-term genomic stability of species throughout megaevolution.

This study aims to determine whether the unique structural features of the highly variable PE_PGRS genes of *Mtb* can explain the uneven distribution of spontaneous mutations across the genome.

In this study, we explore the hypothesis that the primary structure of PE_PGRS rich with sterically active tetramers such as CGGC and deficient with out-of-frame stop codons predispose them to mutations. Programmed DNA variability, as exemplified by *Mtb,* may represent a conserved evolutionary strategy among all DNA-based life forms.

## Results

### Elevated abundance of secondary-structure-forming CGGC motifs in PE_PGRS genes

The WHO catalog of drug resistance-associated mutations in *Mtb* includes over 17,000 variants across multiple genes^34^, linked to resistance to first- and second-line anti-TB drugs. Comprehensive cataloging of mutations in drug resistance-associated genes reveals that not all nucleotides are involved in mutagenesis, and strikingly few synonymous (silent) mutations are observed, despite their functional neutrality under natural selection. The long-term use of diagnostic tools such as Xpert® MTB/RIF assay (Cepheid, USA) and GenoType® MTBDRplus assay (Hain Lifescience, Germany) for the detection of mutations associated with resistance to rifampicin and isoniazid further supports the observation that mutations associated with anti-TB drug resistance are restricted to specific nucleotide positions, and neighboring nucleotides around resistance-associated mutation hotspots are seldom involved in mutagenesis.

**Fig. 1:**
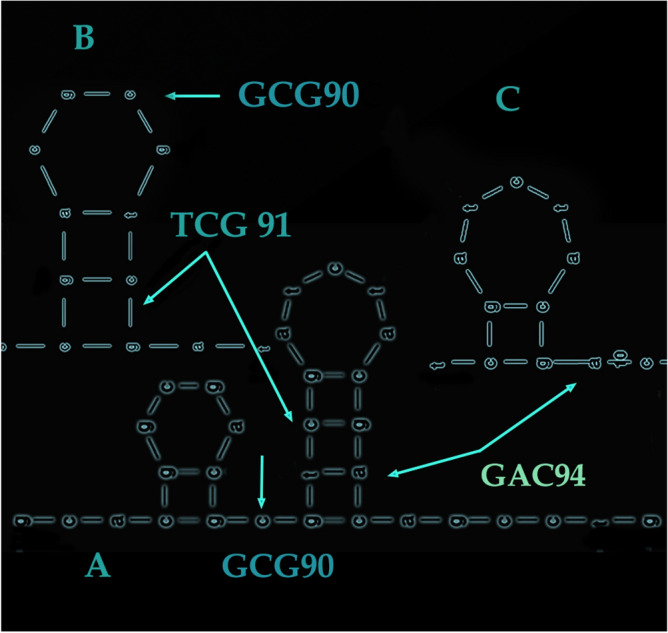
Predicted secondary DNA structures in the ***gyrA*** region encompassing codons 88–94 demonstrate alternative hairpin topologies and interaction energies that may influence the spectrum of quinolone-resistance mutations (Ggc88Tgc, gCg90gTg, Tcg91Ccg, gAc94gGc, Gac94Aac, gAc94gTc, Gac94Cac) (Mfold; Zuker, 2003). (**A**) – Hairpins in the locus carrying codons 90, 91, and 94 (Energy: ΔG = –3.19, ΔH = –43.10, ΔS = –128.68, Tm = 61.8 °C); (**B, C**) – Hairpins with alternative secondary structure topologies carrying codon 90 (Energy: ΔG = –2.61, ΔH = –32.00, ΔS = –94.76, Tm = 64.5 °C) and codon 94 (Energy: ΔG = –0.99, ΔH = –22.70, ΔS = –70.00, Tm = 51.1 °C).

The design of probes targeting single nucleotide polimorphism in anti-TB drug resistance loci, using secondary DNA structure prediction tools such as UNAFold (Quikfold)^35^, reveals that mutations frequently occur at sites prone to hairpin formation. Changes in Na^+^ and Mg^2+^ concentrations, as specified in the Quikfold settings, can alter predicted hairpin structures in silico (Fig. 1, Suppl. Tab. 1). The effectiveness of Quikfold has been validated by its application in predicting hairpin structures and PCR annealing temperatures for diagnostic oligonucleotides. Conformational analysis of Mtb mutation sites associated with drug resistance reveals key sequence motifs—including GCCG, CGGC, GCGC, GGG, GGGG, and CTGC—that play a significant role in ssDNA hairpin formation. DNA secondary structures may redistribute local interaction energies and charges, which can lead to nucleotide misincorporation and replication-associated mutagenesis.

In the *Mtb* genome, only GGG, CGGC, and GCCG among the 28 studied k-mers participating in the formation of DNA conformational structures showed elevated genome-wide densities (~ 1.6 motifs per 100 nt each); for the others, dencity remained below 1 motif per 100 nt. Within PE_PGRS genes, tetramer abundance > 2 per 100 nt was recorded for AACG, GCGC, GCCG, GGG, and CGGC, but frequencies ≥ 4 motifs per 100 nt were limited to GGG (10 PE_PGRS genes) and CGGC (50 PE_PGRS genes, including 30 with ≥ 5 motifs per 100 nt) (Fig. 1).

**Fig. 2:**
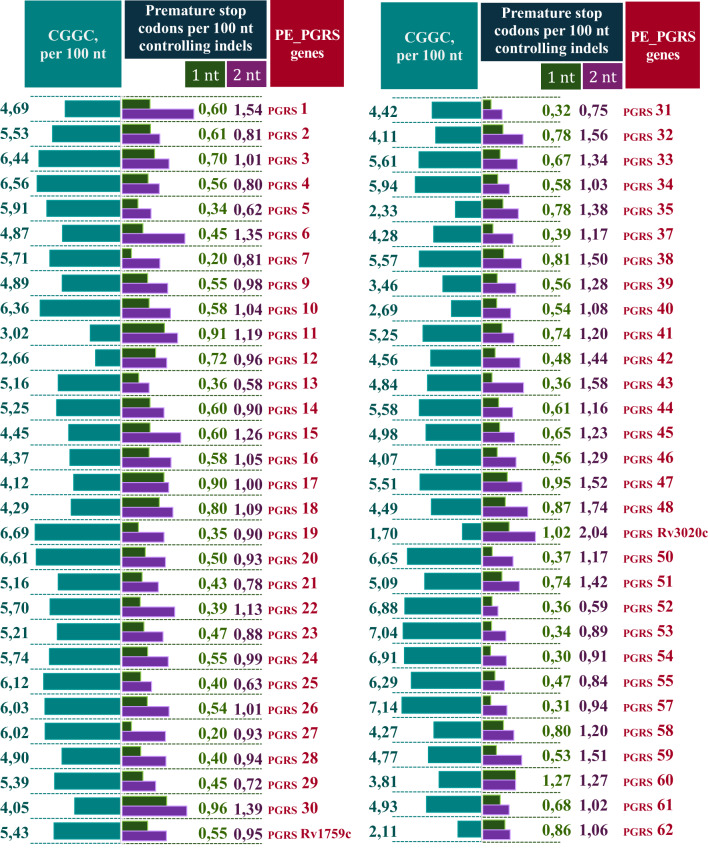
(**a, b**) Abundance of CGGC and pre-stop codons (TAG, TGA, TAA), controlling tolerance to 1nt and 2nt frameshift mutations in PE_PGRS genes. In PE_PGRS genes, Spearman’s correlation coefficients indicate a weak but significant inverse correlation with the frequency of 1-nt pre-stop codons (Spearman’s r = –0.257, n = 61, *p* = 0.045) and a non-significant very weak correlation with 2-nt pre-stop codons (Spearman’s r = –0.08, *n* = 61, *p* = 0.4937). Binomial test showed significant over-representation of CGGC in PE_PGRS genes in the CG-rich model (p = 1.1 × 10^−3^ for ≥ 4 CGGC repeats) and in the Gly-Gly-rich model (*p* = 0.035 for ≥ 5 repeats).

PE_PGRS genes exhibited significantly higher CGGC tetramer density (per 100 nt) (mean 4.97, range 1.7–7.4; 95% CI: 3.63–6.33) compared to the genome-wide mean (1.62; 95% CI: 0.97–2.27; Mann–Whitney U test: U = 219,210, Z = 12.70, p < 10^–36^) (Fig. 2). In contrast to PE_PGRS genes, several genes in the *Mtb* genome are completely devoid of the CGGC tetramers, indicating a non-random distribution. These include *Rv0011c* (cell division protein CrgA), Rv0616A (antitoxin VapB29), *Rv0722* (50S ribosomal protein L30), *Rv1038c,* *Rv1197,* and *Rv2347c* (ESAT-6–like proteins EsxJ, EsxK, EsxP), *Rv3053c* (glutaredoxin electron transport protein NrdH), *Rv3320c* (ribonuclease VapC44 toxin), and a group of genes encoding conserved or hypothetical proteins—*Rv0460,* *Rv2452c,* *Rv3196A,* *Rv3678A,* *Rv2722*, *Rv0100*. Thus, CGGC enrichment in PE_PGRS genes may promote secondary structure formation and create mutational hotspots.

Although PE_PGRS proteins are highly enriched in glycine residues, which are encoded by GGG, GGA, GGC, and GGT codons, the expected probability of forming a CGG–CGG junction under a random Gly–Gly model is 0.0625, yielding an expected CGGC abundance of ~ 2 motifs per 100 nt. Under a conservative GC-bias and codon-usage null model, CGGC frequency is expected to be ≤ 2 motifs per 100 nt. In contrast, a substantial proportion of PE_PGRS genes (50/62) exhibit 4–7 motifs per 100 nt, significantly exceeding this expectation (exact binomial test, p ≈ 1.1 × 10^−3^ for ≥ 4 motifs). This over-representation indicates that CGGC enrichment in PE_PGRS genes cannot be explained by glycine content or GC bias alone.

To determine whether the absence of CGGC motifs is attributable to gene length, we analyzed 281 small genes (≤ 297 nt, comparable in length to two PE_PGRS genes). CGGC motifs occur even in very short genes: the shortest annotated gene (93 nt) contains one motif. Across genes ≤ 297 nt, the mean CGGC density is 1.46 per 100 nt (range 0.34–5.41). Linear regression showed no relationship between gene length and CGGC abundance (ANOVA: R^2^ = 0.00049, F(1,279) = 0.136, *p* = 0.712). Thus, the absence of CGGC in some small genes cannot be explained solely by their length.

### Inter-species and intra-genomic comparisons of the abundance of CGGC tetramers

**Fig. 3:**
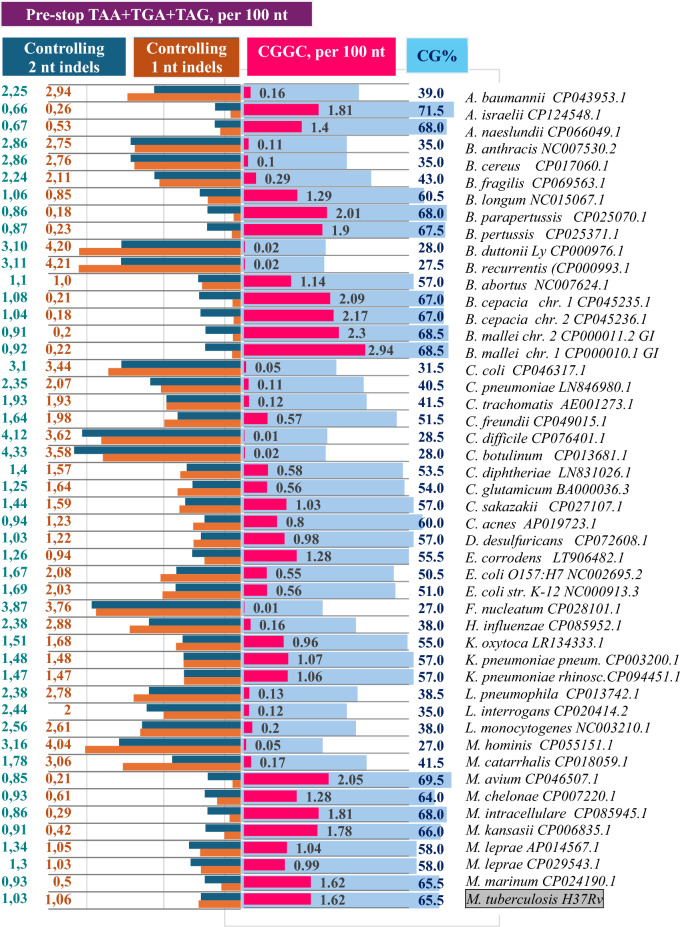

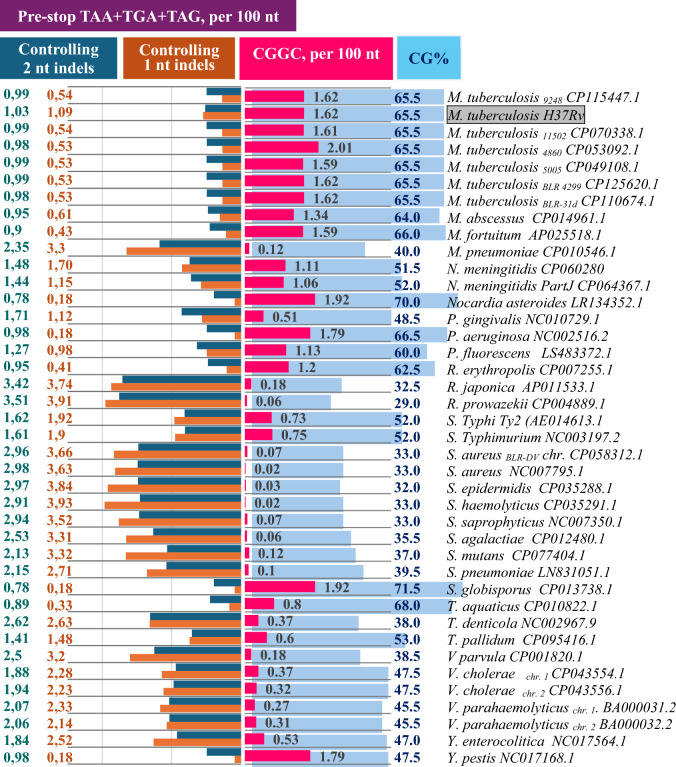
Inverse correlation between CGGC tetramer abundance and pre-stop codons (TAA, TGA, TAG) controlling tolerance to 1 nt and 2 nt frameshift mutations across 87 bacterial genomes with different total GC content (%). Values represent genome-wide mean frequencies of CGGC tetramer, 1-nt pre-stop codons, and 2-nt pre-stop codons (per 100 nt) calculated across all coding sequences. Spearman’s correlation coefficients indicate a weak but significant inverse correlation between CGGC density and pre-stop codons controlling tolerance to 1 nt (Spearman’s r = –0.207, *n* = 3749, p = 1.226 × 10^−37^) and 2 nt (Spearman’s r = –0.162, *n* = 3749, p = 9.837 × 10^−24^) frameshift mutations. This pattern is observed not only in PE_PGRS genes and ***Mtb genome*** but also across taxonomically diverse species, including closely related bacteria within the same genus. The observed distribution cannot be explained solely by variation in genomic G + C content.

**Fig. 4:**
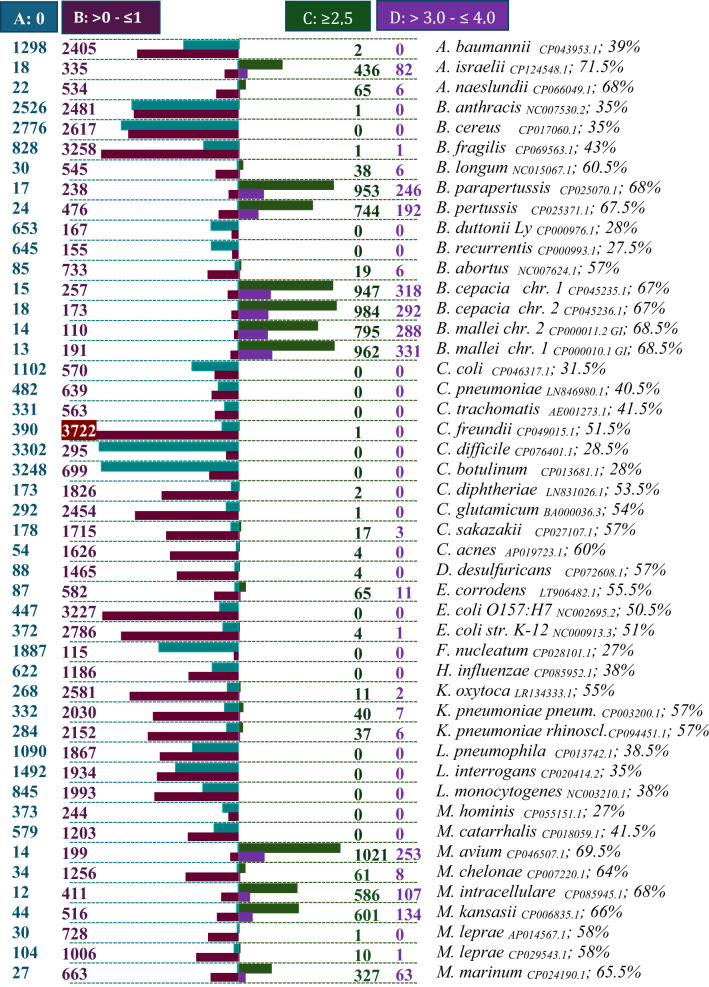

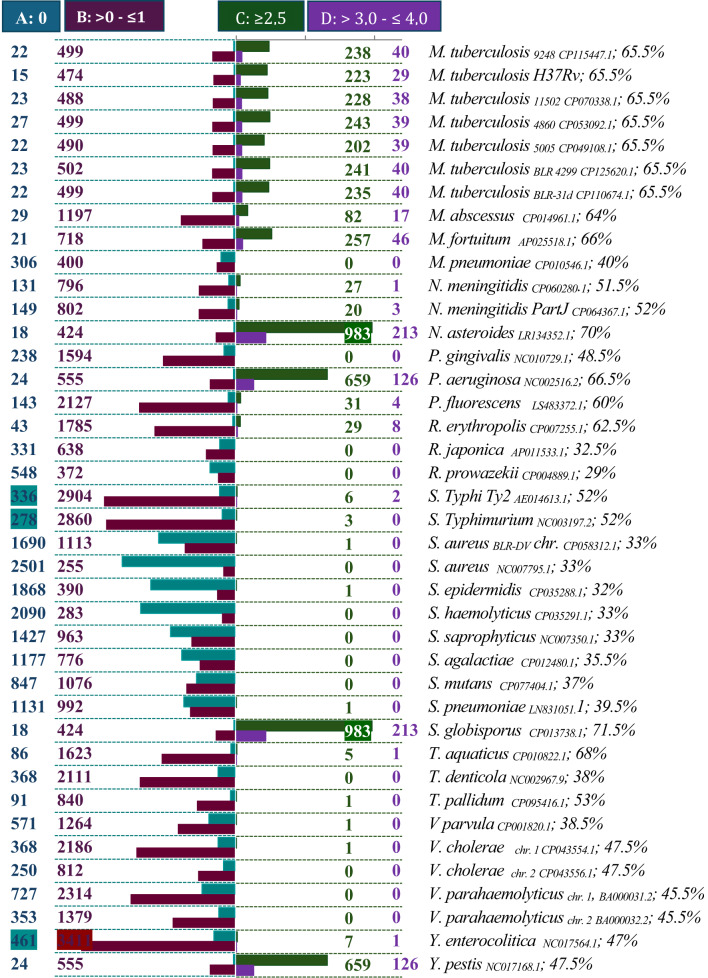
Number of genes in microbial genomes categorized by CGGC tetramer abundance: (**A**) genes without CGGC; (**B**) genes with CGGC abundance > 0 to ≤ 1 per 100 nt; (**C**) genes with CGGC abundance ≥ 2.5 per 100 nt; (**D**) genes with CGGC abundance > 3.0 to ≤ 4.0 per 100 nt

The distribution of CGGC tetramers was highly variable across bacterial taxa and within the *Mtb* genome, and was not determined solely by genomic G + C contentc (Figs. 3 and 4). Within genera, closely related species often differed markedly despite identical G + C%: *Yersinia pestis* vs *Y. enterocolitica* (1.79 vs 0.53 per 100 nt; genes with ≥ 2.5 CGGC: 659 vs 7; p < 0.01, Mann–Whitney U test), *Pseudomonas aeruginosa* vs *P. fluorescens* (1.79 vs 1.13; 659 vs 31), and *Klebsiella pneumoniae* (1.06–1.07; 37–40) vs Salmonella enterica (0.73–0.75; 3–6) and *E. coli* (0.55–0.56; 0–4). *Neisseria meningitidis* showed higher CGGC levels (1.06–1.11; 20–27) than E*. coli* and Salmonella despite comparable G + C. Among mycobacteria, CGGC density in *Mtb* (1.62 per 100 nt, number of genes with CGGC ≥ 2.5: 202–243) was higher than in *M. leprae* (0.99–1.04; 1–10), and lower than values in *M. avium*, *M. intracellulare*, *M. kansasii* (2.05, 1.85, 1.78; 1021, 586, 601). *Corynebacterium diphtheriae*, a conserved actinobacterial pathogen and the causative agent of diphtheria, exhibits an even lower CGGC content (0.58 per 100 nt) than *M. leprae* and most mycobacteria or opportunistic actinomycetes. High average CGGC density is observed in *Bordetella pertussis* and *B. parapertussis* (1.90 and 2.0; 744 and 953).

Importantly, most of the studied microorganisms harbor gene pools completely devoid of CGGC tetramers. In mycobacteria, this fraction ranges from 12 to 104 genes per genome (*Mtb:* 15**–**27 genes; *M. leprae:* up to 104 genes) (Fig. 4).

Analysis of the prevalence of gene pools with varying CGGC content across bacterial species reveals that genes with moderate enrichment (> 2.5– ≤ 3 per 100 nt) are generally more widespread than those with higher enrichment (> 3– ≤ 4 and > 4 per 100 nt). However, the size of the high-CGGC gene pool (> 3 per 100 nt) varies markedly across species: 68–93 genes in *Mtb,* substantially extended in *M. avium* subsp. *avium* (253), *M. intracellulare* (115), and *M. kansasii* (216), and remains limited in *M. chelonae* (8), *M. leprae* (1), and *M. abscessus* (17). Other bacteria with a significant pool of highly CGGC-enriched genes (> 3 per 100 nt) include *Bordetella pertussis* (264 genes) and *B. parapertussis* (205), *Burkholderia mallei* (380) and *B. cepacia* (312), *Streptomyces globisporus* (227), and *Nocardia asteroides* (227). Closely related species may exhibit markedly different representation of genes highly enriched in CGGC (> 3 per 100 nt): *Yersinia enterocolitica* (5 genes) versus *Y. pestis* (130), *Pseudomonas fluorescens* (4) versus *P. aeruginosa* (130), and *Actinomyces naeslundii* (6) versus *A. israelii* (93). The apparent heterogeneity of genes by the degree of their CGGC abundance may reflect variation in mutational potential across genes within a genome and between genomes of different bacterial species.

### CGGC-induced hairpins as catalysts of replication errors

The CGGC tetramer exhibits structural advantages that may underlie its promutagenic potential. Thermodynamic modeling (mfold^35^) of DNA hairpins with identical loops but varying stem compositions showed that CGGC forms the most stable hairpin among tested sequences (ΔG = –4.33 kcal/mol, highest Tm), followed by CGGG, CGCG, GGG, GGGG, and non-GC-rich controls (Suppl. Tab. 2).

Compared to GGG/CCC, CGCG, and GCGC motifs, CGGC provides greater stem stability and more pairing options (including the inverse GCCG and submotifs CCG and GCC, with GCC yielding more stable base pairing). This enhanced stability may better counteract loop destabilization and redistribute energy more efficiently during replication, thereby increasing the likelihood of nucleotide misincorporation and mutagenesis.

Notably, CGGC tetramers are present within major resistance-associated sites in *katG* (Ser315) and *gyrA* (near Ala90 and Ser91), and are frequently detected near mutation sites in PE_PGRS genes from sequenced *Mtb* strains (Suppl. Tables 1 and 2). Their presence in these loci indicates that CGGC may shape local sequence architecture, promoting mutations by disrupting replication fidelity via secondary structure formation.

### Evolutionary divergence in the abundance of out-of-frame stop codons conferring 1-nt and 2-nt frameshift tolerance across bacteria

A one- or two-nucleotide insertion or deletion within a coding sequence typically induces a frameshift, potentially resulting in a premature stop codon and the production of a truncated, non-functional peptide. A low abundance of premature stop codons (PSC)—TAA, TGA, TAG—allow translation to proceed far downstream of the original frameshift site, conferring a degree of frameshift tolerance. This phenomenon was observed in the *PE_PGRS2* gene of the *Mtb* strain 4860, in which a premature stop codon appears at position 1001 nt, despite the primary deletion occurring at position 591 nt. A low abundance of premature stop codons may lead to the loss of the canonical stop codon and extension of the protein into intergenic DNA regions, as observed for *PE_PGRS12* in *Mtb* strain 5005 and *M. bovis* BCG Pasteur (Suppl. Fig. 1).

We analyzed the abundance of PSC within PE_PGRS genes of *Mtb,* as well as in the rest of its genome, and across a range of other bacterial species. Our analysis revealed that PE_PGRS genes with elevated CGGC content tend to exhibit a lower frequency of PSC, terminating translation upon a 1- or 2-nt frameshift mutations.

In PE_PGRS genes, CGGC abundance displayed a weak but significant negative correlation with the frequency of PSCs controlling 1-nt frameshift mutations (Spearman’s ρ = –0.257, n = 61, p = 0.045) and a very weak, non-significant negative correlation with PSCs controlling 2-nt frameshift mutations (Spearman’s ρ = –0.08, n = 61, *p* = 0.494) (Fig. 2).

Multiple linear regression showed that GC content was the dominant predictor of PSC frequency (partial r =  − 0.557), while CGGC density had a weaker, marginally non-significant effect (partial r =  − 0.233, β =  − 0.052, *p* = 0.070). Thus, the reduction in AT-rich stop codons is largely a passive consequence of GC pressure rather than CGGC accumulation.

An inverse correlation between CGGC and PSC abundance controlling 1-nt (Spearman’s ρ = –0.207, n = 3751, p = 1.23 × 10^−37^) and 2-nt (Spearman’s ρ = –0.162, *n* = 3751, p = 9.84 × 10^−24^) frameshift mutations, typical of the *M. tuberculosis* genome, was also observed across diverse bacterial species (Fig. 3).

### Representation of the gene pool lacking PSCs controlling 1-nt and/or 2-nt frameshift tolerance across bacteria

A reduced abundance of PSCs may impair the efficiency of cellular control over the consequences of frameshift mutations and may serve as a genetic mechanism enabling abrupt changes of protein properties which may be important for antigenic variation and protein diversification. Reduced density of PSCs in the genome may promote mutagenesis and act as a promutagenic factor encoded in the DNA, contributing to rapid protein evolution.

**Fig. 5:**
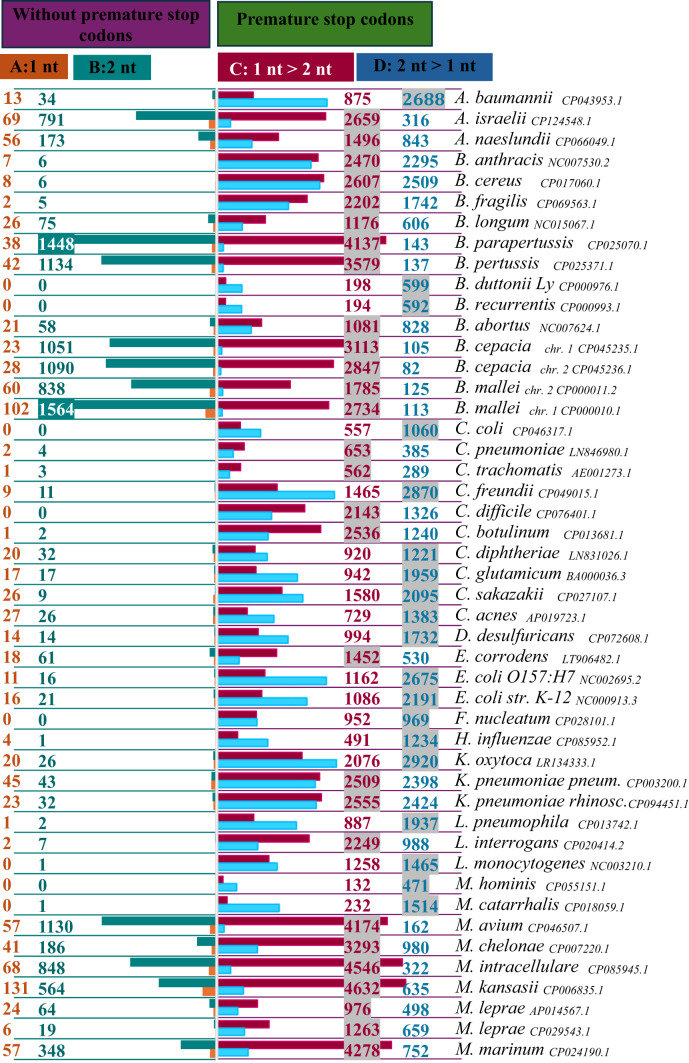

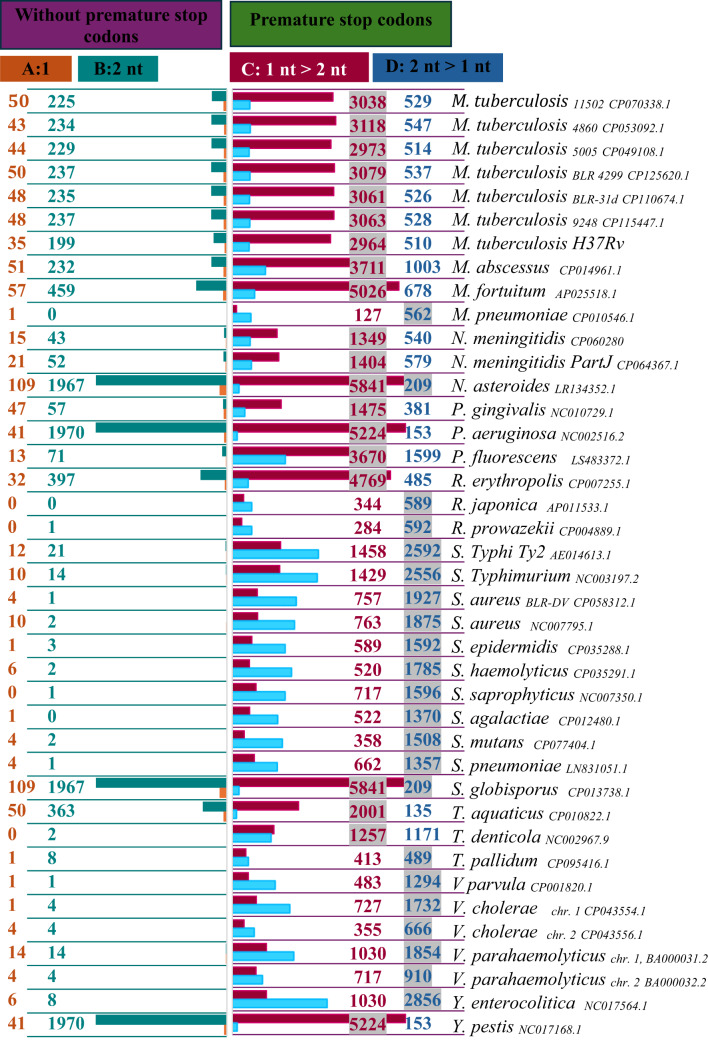
Number of genes in microbial genomes categorized by abundance of pre-stop codons, controlling 1 nt and 2nt frameshift mutation tolerance: (**A**) genes without pre-stop codons, controlling 1nt; (B) genes without pre-stop codons, controlling 2 nt; (**C**) genes with 1nt > 2nt; (**D**) genes with 2 nt > 1nt.

*Genomic abundance of genes lacking PSCs conferring both 1- and 2-nt frameshift tolerance. Mtb* harbors genes completely devoid of PSCs controlling both 1-nt and 2-nt frameshift tolerance (e.g., Rv0064A, Rv1434, Rv1590, Rv2774c, Rv1473A (transcriptional regulator), Rv2056c (30S ribosomal protein S14)). Such gene pools were detected in nearly all studied microbial genomes (Figs. 4 and 5), ranging from 0 to 102 genes per genome (mean 26.3). Notably larger pools were observed in *A. israelii*, *A. naeslundii*, *B. parapertussis*, *B. pertussis*, *B. mallei* (both chromosomes), *K. pneumoniae subsp. pneumoniae*, most mycobacteria (except *M. leprae*), *N. asteroides*, *Porphyromonas gingivalis*, *P. aeruginosa*, *Rhodococcus erythropolis*, *S. globisporus*, *Thermus aquaticus*, and *Y. pestis.*

*Distribution of genes lacking PSCs conferring 1-nt or 2-nt frameshift tolerance.* Genes lacking PSCs exclusively for 1-nt frameshift tolerance were considerably more numerous (e.g., 1,148 in *B. parapertussis*, 1,564 in *B. mallei* chr. 1, and up to 1,967–1,970 in N*. asteroides*, *S. globisporus*, *Y. pestis*, and *P. aeruginosa*). The average number of genes without PSCs controlling 2-nt frameshift tolerance across microorganisms was approximately 264.3. Among mycobacteria, the largest gene pools lacking PSCs for 1-nt frameshift tolerance were found in *M. avium* (1,130 genes), *M. intracellulare* (848 genes), *M. kansasii* (564 genes), and *M. fortuitum* (459 genes). In *Mtb*, the number of genes lacking PSCs for 2-nt frameshift tolerance ranged from 199 to 237, similar to that in *M. abscessus* (232 genes).

### Frameshift tolerance varies across genes and species

The abundance of PSCs, conferring 1-nt frameshift tolerance, ranged (per 100 nt) from 0.18 in *Y. pestis*, *B. parapertussis*, *B. cepacia,* and *S. globisporus* to 4.21 in *Borrelia recurrentis* (mean 1,77); for 2-nt frameshifts, this value ranged from 0.66 in *A. israeli* to 4.33 in *C. botulinum* (mean 1,78), depending on the species. In *Mtb*, the average abundance of PSCs conferring 1-nt and 2-nt frameshift tolerance was 0.53–0.54 (1.09 in the *Mtb* H37Rv strain), and 0.98 (1.03% in *Mtb* H37Rv), respectively, while in *M. leprae*—obligate pathogen and obligate intracellular parasite─these levels were higher: 1.03–1.05 and 1.3–1.34, respectively.

Control over 1-nt and 2-nt frameshift tolerance varies both between genes and across species: some genes and bacterial taxa exhibit a predominance of 1-nt frameshift control, while others show dominance of 2-nt frameshift control. Among the 74 bacterial species analyzed, 33 showed genome-wide dominance of 1-nt frameshift control, whereas 36 were dominated by 2-nt frameshift control (Fig. 5).

Mycobacteria exhibited stronger genomic robustness to 1-nt than to 2-nt frameshifts, as observed in *Mtb (*2,964–3,079 vs. 510–547 genes), *M. fortuitum* (3,028 vs. 5,026), *M. chelonae* (1,646 vs. 3,293), *M. abscessus* (1,945 vs. 3,711), *M. intracellulare* (2,994 vs. 4,546), *M. avium* subsp. *avium* (2,679 vs. 4,174), *M. kansasii* (2,918 vs. 4,632), and *M. leprae* (541 vs. 1,263).

Certain genes (such as PE_PGRS) and certain microbial species (*Mtb* and non-tuberculosis mycobacteria, *Y. pestis*, *B. pertussis*, *B. cepacia, S. globisporus, N. asteroids, P. aeruginosa*) may have increased adaptive potential due to attenuated control over 1-nt and/or 2-nt frameshifts.

## Discussion

Earlier, it was presumed that the proper functioning of bacteria is encoded in their genome at multiple hierarchical levels, each constrained by specific physical forces^36^. Variations in tetranucleotide frequencies were suggested as an indicator of the structural organization of microbial genomes^37^.

To explore this further, we conducted a genome-wide analysis of CGGC tetramer abundance across mycobacteria and diverse taxa of bacteria. We hypothesized that these motifs could be associated with genomic regions of high plasticity.

We focused on the highly variable PE_PGRS genes of Mtb. Our analysis revealed that these genes are significantly enriched in CGGC tetramers relative to the genomic average and markedly depleted in PSCs, particularly those associated with robustness to 1-nt frameshifts.

In silico conformational analyses of DNA at mutation sites associated with resistance to anti-TB drugs predicted the formation of short hairpin structures (7–13 nt in length), with stems frequently containing CGGC or related tetramers (e.g., CGCG, GCGC, CCGG).

Based on these observations, we propose a model wherein the overrepresentation of such sterically active motifs (e.g., CGGC) could create a genomic environment that facilitates nucleotide misincorporation: during replication secondary structures formed by sterically active motifs (CGGC or other) —can alter local electrostatic, hydrophobic interactions, potentially compromising replication fidelity and thereby contribute to elevated mutation rates in these regions.

Our findings indicate that the uneven distribution of mutational variability in the *Mtb* genome, particularly within hypervariable PE_PGRS genes, correlates with their primary sequence composition (elevated content of specific motifs, including sterically active CGGC tetramers). These findings support a model in which the potential for mutational variability in PE_PGRS genes in Mtb as well as in the genomes of other microorganisms, is encoded within the primary DNA sequence. Genes enriched in CGGC tetramers in *Mtb* (PE_PGRS) may exhibit a significantly higher susceptibility to mutation. However, whether CGGC-rich regions actively promote this variability through secondary DNA structure formation remains to be experimentally validated.

CGGC motifs may facilitate the formation of secondary DNA structures, analogous to telomeric repeats in eukaryotic genomes. The human TTAGGG motif includes the GGG trimer and the out-of-frame stop codon TAG, and contributes to the formation of stable G-quadruplex structures^38^, thereby influencing replication and termination signals in eukaryotes.

Several bacterial species outside the genus Mycobacterium also show relatively high CGGC tetramer abundance combined with low frequencies of PSCs, including *B. parapertussis*, *B. pertussis*, *B. cepacia*, *B. mallei*, *N. asteroides*, *P. aeruginosa*, *S. globisporus*, *Y pestis*, *A. israelii*, and some non-tuberculosis mycobacteria, but not *M. leprae*. Species within the same genus that differ in their clinical relevance and pathogenicity for humans can exhibit significant differences in CGGC and PSC densities. Pathogenic species such as *Y. pestis*, *P. aeruginosa*, and *Mtb* possess markedly higher CGGC and lower PSC density compared to *Y. enterocolitica*, *P. fluorescens*, and *M. leprae*. Compared to *M. leprae*, Mtb exhibits higher proportion of genes with CGGC > 3 per 100 nt, which aligns with epidemiological observations of continuing global burden of tuberculosis versus the declining incidence of leprosy—although many other genetic, immunological, and socio-economic factors clearly contribute to these epidemiological differences.

When ranked by decreasing CGGC abundance and increasing PSC frequency, the studied non tuberculosis mycobacteria (NTM) follow the order: *M. avium*, *M. intracellulare*, *M. kansasii*, *M. marinum*, *Mtb*, *M. fortuitum*, *M. abscessus*, *M. chelonae*, and *M. leprae*. Interestingly, this order correlates with ethiological structure of NTM infections, in which *M. avium*, *M. intracellulare* dominate over other NTM.

In recent years, NTM have demonstrated remarkable adaptive capabilities. In several regions, reported rates of NTM infections now exceed those of tuberculosis, with increasing cases in apparently immunocompetent children and adults and evidence of person-to-person transmission. The drivers of this rise are only beginning to be elucidated^39^. Differences in virulence and pathogenesis between *Mtb* and NTM may arise from variations in virulence determinants, such as cord factor and the type VII secretion system. However, the ability of NTM to persist within the host, evade immune responses, and resist antimicrobial treatment may be partly explained by a substantial gene pool enriched in CGGC tetramers and depleted in premature stop codons. This combination may confer enhanced mutational plasticity, thereby amplifying the adaptive potential of NTM. High CGGC abundance coupled with low premature stop codon density may drive evolution by increasing mutational plasticity in specific gene subsets. These features could underlie the observed differences in adaptive potential and epidemiological success among pathogens, including NTM.

The results obtained in this study advance the concept of DNA as a dual-information carrier: digital information defines protein structure via the genetic code, while analogue information reflects sequence-dependent physicochemical properties of DNA that promote the formation of secondary structures, potentially influencing the fidelity of replication and repair processes. PE_PGRS genes were shown to be evolutionarily optimized to exploit this analogue layer, which allows them to exhibit enhanced variability.

The obtained data challenge the canonical concept of random spontaneity of mutations and instead support a model of “constrained spontaneity” for mutations. While mutations are often described as purely random, our findings indicate that their likelihood is not uniform across the genome. We hypothesize that spontaneous mutations emerge more frequently in specific “turbulence zones” – genomic areas enriched with sterically active motifs like CGGC, which may be prone to conformational instability. An elevated CGGC abundance in specific genes may enhance their evolutionary plasticity; however, even a single CGGC motif (or another sterically active sequence) may be sufficient to trigger mutational events. Thus, what is often considered “spontaneous” may be influenced by the analog information embedded in the DNA sequence.

Secondary structure formation in single-stranded DNA is a flexible process, influenced by many factors, including ion concentration and temperature, and is not a strictly fixed process, which predetermines the spontaneous nature of mutations. Analysis of conformational structures at mutation sites indicates that their formation is a universal process, found in the genomes of bacteria, viruses, and human cells (Suppl. Tab. S1).

Although the replication machinery is generally highly accurate, ensuring the megaevolutionary stability of genomes, certain loci remain structurally predisposed to replication errors and reduced efficiency of repair mechanisms. DNA loci harboring CGGC motifs that form stable hairpins can stall replication forks and reduce the fidelity of both DNA polymerases and repair enzymes, increasing mutation fixation rates. These hairpins, stabilized by strong base-pairing interactions, can resist strand separation and efficient melting and coating by single-stranded DNA-binding proteins (SSBs)^40,41^.

As previously shown^41^, pathogenic microorganisms can adjust their mutation rates. Mutator strains emerge and persist within bacterial populations at varying prevalence. Our data on the CGGC tetramer densities and PSCs were compared with the frequency of mutator strains in bacterial populations. Microorganisms with higher mutator frequencies, such as *P. aeruginosa* (19.5–92%) and *N. meningitidis* (22.2–56.8%), exhibited higher CGGC content (1.79% and 1.11%, respectively), whereas species with low mutator rates, including *S. aureus* (1.4–14.6%) and *Vibrio parahaemolyticus* (0–5.13%), showed lower CGGC levels (0.07 and 0.27, respectively) (Suppl. Tab. S3).

Our findings support the idea that the genetic code, through its degeneracy, governs the composition of the primary nucleotide sequence, thereby modulating the physicochemical properties of DNA to influence genomic stability—by minimizing secondary DNA structures—or, conversely, to enhance local mutability via sterically active tetramers.

The influence of DNA k-mers (e.g., CGGC) on mutability, as revealed by our study, has implications for genome engineering. Since modulating nucleotide composition via editing can inadvertently alter local DNA stability and mutation rates, the analog architecture of DNA must be accounted for in genome editing design. Identifying motifs that predispose DNA to form secondary structures will enable genome engineering with controlled stability.

PE_PGRS genes of *Mtb* display approximately threefold higher mutational variability than non-PE/PPE genes, driven by non-synonymous substitutions and frameshift-inducing indels, and occurring largely under near-neutral selection (dN/dS ≈ 0.87). Several mechanisms have been implicated in elevated mutagenesis in *Mtb,* including mutations in *gyrA* affecting DNA gyrase activity^46,47^ and lineage-specific alterations in DNA repair and recombination (3R) systems^45^, both of which are expected to increase mutation rates genome-wide but do not explain the locus-specific increase in mutational burden observed in PE_PGRS genes. In contrast, the presence of CGGC repeats defines a distinct sequence architecture that promotes elevated mutational plasticity specifically within PE_PGRS loci. Recent studies of RNA G-quadruplexes (rG4s) in mycobacteria have shown that PE_PGRS genes exhibit an rG4 density more than tenfold higher than that observed across the full set of *Mtb* transcripts. For rG4s, as for CGGC motifs, no correlation was detected between glycine content and rG4 density in PE/PPE transcripts^49^.

Our findings provide new insights into the role of PSCs. While the prevailing hypothesis posits that gene sequences are enriched with PSCs to promptly terminate translation upon frameshifts and minimize energy and resource expenditure^43^, our data suggest a more complex context. The control of robustness against 1-nt and 2-nt frameshift mutations appears to be less stringent in certain genomic regions. Across all analyzed genomes, we identified gene subsets lacking PSCs associated with 1-nt or 2-nt frameshift mutation control, as well as subsets entirely devoid of PSCs. Furthermore, in the studied microorganisms PSC density varied markedly among different gene pools, indicating diverse genomic strategies for managing frameshift errors. The absence of PSCs decouples frameshift mutations from early translational termination and translation to continue beyond frameshift mutations. This increases the coding potential of the genome by allowing production of altered polypeptides in which functionally critical regions may retain activity, thereby reducing the selective cost of frameshift events^50^. Markedly reduced PSC density may represent a genetic mechanism for altering PE_PGRS protein composition and extending protein length through incorporation of intergenic sequences when canonical stop codon is displaced from frame, as observed for PE_PGRS12 in BCG vaccine strains and the *Mtb* strain 5005 (Suppl. Fig. 1), that may potentially contribute to antigenic drift and immune evasion. This reduction in PSC density may also constrain complete replacement of AT with GC under GC bias pressure, as unrestricted CG enrichment (including CGGC motifs) leads to automatic depletion of AT-rich codons, including premature stop codons (TAA/TAG/TGA). Low PSC density in certain gene subsets may reduce frameshift robustness and permit synthesis of proteins with modified composition and length.

Mutational plasticity in *Mtb* and other bacterial species may not arise solely from external selective pressures or random replication errors, but could be intrinsically encoded in the genome. In Mtb, we identified a substantial gene set with elevated mutational plasticity, characterized by high CGGC density and reduced PSC abundance. This genomic architecture positions *Mtb* as a pathogen with high adaptive potential, where primary DNA sequences in certain genes exhibit pro-mutagenic properties. A similar gene pool enriched in CGGC tetramers and depleted in PSCs was also found in other successful pathogens, including *P. aeruginosa*, *Y. pestis*, *A. israelii*, *B. pertussis*, *B. parapertussis*, *N. asteroides*, and *B. cepacia*.

Collectively, our findings support the dialectical hypothesis of evolution driven by a dynamic balance between adaptive mutagenesis and genomic stability^38^: while CG-rich regions maintain DNA stability and replication fidelity, conformationally active CGGC tetramers in ssDNA serve as localized mutation hotspots. These structures disrupt replication fidelity through secondary conformational changes, altering electrostatic and hydrophobic interactions and increasing nucleotide misincorporation.

### Limitations

This study focuses on the potential link between CGGC tetramers, DNA mutability and elevated variability of PE_PGRS genes in *Mtb,* and it has several limitations: it is restricted to a single CG-rich tetramer (CGGC) and a single gene family (PE_PGRS) within *Mtb*. Predicted secondary structures formed by CGGC motifs were not experimentally validated in this work. However, prior studies from our group demonstrated that conformational structures significantly affect the melting temperature (Tm) of dual diagnostic PCR probes designed to detect polymorphisms in anti-TB drug resistance genes (*gyrA*, r*poB*, *rrs*, *katG*) and genotypic markers (*Rv2994*, *pstA1*, *Rv1956*, *mce3B*, *Rv0557*, *mce3F*, *rpoC*, *pncA*). The proposed association between CGGC enrichment and DNA mutability relies on comparative genomic analyses rather than direct experiments. Despite these constraints, the large-scale dataset supports a relationship between CGGC density and regional variation in PE_PGRS genes.

## Methods

### Whole-genome sequencing

All experimental procedures involving live *Mtb* cultures were performed in a certified Biosafety Level 3 (BSL-3) laboratory in accordance with institutional and national biosafety guidelines.

High-throughput sequencing of *M. tuberculosis* genomes was performed using MiSeq (Illumina) and MinION (Oxford Nanopore) platforms. Mycobacterial DNA was isolated using the QIAGEN Blood & Cell Culture DNA Maxi Kit (Cat. No./ID: 13,362) or the QIAamp DNA Mini Kit (Cat. No./ID: 51,304). Genome assemblies were generated with SPAdes v3.14, A5-miseq v20160825, Flye v2.7b, and Canu v1.8. The obtained complete genome sequences were deposited in GenBank: *M. tuberculosis* 11,502 (CP070338.1, BioSample SAMN17832565), *M. tuberculosis* 5005 (CP053092.1, SAMN14150054), *M. tuberculosis* 4860 (CP049108.1, SAMN14598146), *M. tuberculosis* 31d (CP110674.1, SAMN31644110), *M. tuberculosis* 4299 (CP125620.1, SAMN32350377), and *M. tuberculosis* BLR 9248 2019 (CP115447.1).

### Bioinformatics research

Thermodynamic modeling of DNA secondary structures was carried out using the Quikfold web-based tool (UNAFold Web Server; Zuker, 2003). To assess the contribution of specific stem motifs to hairpin stability, a set of oligonucleotides was in silico generated with identical loop sequences and variable stem regions. The tested combinations included representative CG-rich configurations observed in Mtb genes, as well as alternative CG arrangements used for comparison. Melting temperatures (Tm) and Gibbs free energies (ΔG) were computed in silico for the following sequences (stem regions in brackets): AA[CGGC]ACCA[GCCG]AA; AA[CGGC]ACCA[GCC]AA; AA[CGGG]ACCA [CCCG] AA; AA[GGGC]ACCA[GCCC]AA; AA[CGGC]ACCA[CCG]AA; CAA[GGG]ACCA [CCC]AAG; AA[GGG]ACCA[CCC]AA; AA[GGG]ACGCGTACA[CCC]AA; AA[GGGG] ACCA[CCCC]AA; CAA[CGG]ACCA[CCG]AAG; A[CTTC]ACCA[GAAG]A (control); A[GTTG]ACCA[CAAC]A (control).

The abundance of nucleotide motifs involved in the formation of secondary conformational structures (GCCGGTGTTG, CGGCGGCAA, AAAA, TTTT, GTCGGC, GCCGAC, TTCT, TAA, CAGA, CCCC, TAG, GTCGG, TCTG, AACC, GGGG, TTGG, GGTT, CCAA, AACG, CGTT, TCGT, GCCGA, ACGA, AGCG, CGCT, GTCG, GCGC, TGA, GGG, CGGC, and GCCG) was assessed across all coding sequences of the *Mtb* genome, including PE_PGRS genes. Genomic analysis of CGGC motifs, and triplets TGA, TAG, and TAA (expressed as percentages or counts per 100 bp) was performed across coding genes of Mtb and compared with bacterial genomes from diverse taxa (88 genomes, 74 species). All genome assemblies processed in this study are available in GenBank (NCBI; Suppl. Tab. 4). Analyses were conducted using java based tool, available on GitHub (https://github.com/ashakirin/microbilogy-2; A. Shakirin, 2022, VMWare Tanzu Labs, Germany).

### Statistical analysis

The CGGC tetramer and PSC densities (per 100 nt) were calculated as the mean for each annotated coding sequence. Statistical analyses, including Shapiro–Wilk normality test, Mann–Whitney U test, Spearman’s rank correlation, linear regression with ANOVA, and binomial tests, were conducted using the online platform StatsKingdom.

## Supplementary Information

Supplementary Information 1.

Supplementary Information 2.

Supplementary Information 3.

## Data Availability

Processed datasets regarding the distribution of CGGC tetramers and TGA, TAG, and TAA out-of-frame stop codons in Mtb and 81 additional microorganisms are publicly accessible via GitHub (https://github.com/Vero-Slizen/Data-for-Scientific-Reports.git). Additional data supporting the findings of this study are available from the corresponding author upon reasonable request.

## References

[1] Hingley-Wilson, S. M., Sambandamurthy, V. K. & Jacobs, W. R. Survival perspectives from the world’s most successful pathogen, *Mycobacterium tuberculosis*. *Nat. Immunol.* **4**, 949–955 (2003). 14515128 10.1038/ni981

[2] Galagan, J. Genomic insights into tuberculosis. *Nat. Rev. Genet.* **15**, 307–320 (2014). 24662221 10.1038/nrg3664

[3] Gagneux, S. Ecology and evolution of *Mycobacterium tuberculosis*. *Nat. Rev. Microbiol.* **16**, 202–213 (2018). 29456241 10.1038/nrmicro.2018.8

[4] Kanabalan, R. D. et al. Human tuberculosis and *Mycobacterium tuberculosis* complex: A review on genetic diversity, pathogenesis and omics approaches in host biomarkers discovery. *Microbiol. Res.* **246**, 126674 (2021). 33549960 10.1016/j.micres.2020.126674

[5] Gordon, S. V. & Parish, T. Microbe profile: *Mycobacterium tuberculosis*: Humanity’s deadly microbial foe. *Microbiology* **164**, 437–439 (2018). 29465344 10.1099/mic.0.000601

[6] Kyu, H. H. et al. Global, regional, and national burden of tuberculosis, 1990–2016: Results from the global burden of diseases, injuries, and risk factors 2016 study. *Lancet Infect. Dis.* **18**, 1329–1349 (2018). 30507459 10.1016/S1473-3099(18)30625-X PMC6250050

[7] Yang, H., Ruan, X., Li, W., Xiong, J. & Zheng, Y. Global, regional, and national burden of tuberculosis and attributable risk factors for 204 countries and territories, 1990–2021: A systematic analysis for the Global Burden of Diseases 2021 study. *BMC Public Health* **24**, 3111 (2024). 39529028 10.1186/s12889-024-20664-w PMC11552311

[8] Chakaya, J. et al. The WHO Global Tuberculosis 2021 Report – Not so good news and turning the tide back to End TB. *Int. J. Infect. Dis.* **124**, S26–S29 (2022). 35321845 10.1016/j.ijid.2022.03.011 PMC8934249

[9] Menzies, N. A. et al. Lifetime burden of disease due to incident tuberculosis: a global reappraisal including post-tuberculosis sequelae. *Lancet Glob. Health* **9**, e1679–e1687 (2021). 34798027 10.1016/S2214-109X(21)00367-3 PMC8609280

[10] Bloom, B. R. A half-century of research on tuberculosis: Successes and challenges. *J. Exp. Med.* **220**, e20230859 (2023). 37552470 10.1084/jem.20230859 PMC10407785

[11] Eldholm, V. & Balloux, F. Antimicrobial resistance in *Mycobacterium tuberculosis*: the odd one out. *Trends Microbiol.* **24**, 637–648 (2016). 27068531 10.1016/j.tim.2016.03.007

[12] Singh, R. et al. Recent updates on drug resistance in *Mycobacterium tuberculosis*. *J. Appl. Microbiol.* **128**, 1547–1567 (2020). 31595643 10.1111/jam.14478

[13] Jones, R. M., Adams, K. N., Eldesouky, H. E. & Sherman, D. R. The evolving biology of *Mycobacterium tuberculosis* drug resistance. *Front. Cell. Infect. Microbiol.* **12**, 1027394 (2022). 36275024 10.3389/fcimb.2022.1027394 PMC9579286

[14] Qian, W. et al. Identification of novel single nucleotide variants in the drug resistance mechanism of *Mycobacterium tuberculosis* isolates by whole-genome analysis. *BMC Genomics* **25**, 478 (2024). 38745294 10.1186/s12864-024-10390-3 PMC11094924

[15] Wei, X. et al. Recent advances and challenges of revolutionizing drug-resistant tuberculosis treatment. *Eur. J. Med. Chem.* **262**, 116785 (2024). 10.1016/j.ejmech.2024.116785 39191032

[16] Swain, S. S., Sharma, D., Hussain, T. & Pati, S. Molecular mechanisms of underlying genetic factors and associated mutations for drug resistance in *Mycobacterium tuberculosis*. *Emerg. Microbes Infect.* **9**, 1651–1663 (2020). 32573374 10.1080/22221751.2020.1785334 PMC7473167

[17] Veyrier, F., Pletzer, D., Turenne, C. & Behr, M. A. Phylogenetic detection of horizontal gene transfer during the step-wise genesis of *Mycobacterium tuberculosis*. *BMC Evol. Biol.* **9**, 1–14 (2009). 19664275 10.1186/1471-2148-9-196 PMC3087520

[18] Xia, X. Horizontal gene transfer and drug resistance involving *Mycobacterium tuberculosis*. *Antibiotics* **12**, 1367 (2023). 37760664 10.3390/antibiotics12091367 PMC10526031

[19] Hirsh, A. E., Tsolaki, A. G., DeRiemer, K., Feldman, M. W. & Small, P. M. Stable association between strains of *Mycobacterium tuberculosis* and their human host populations. *Proc. Natl Acad. Sci. USA* **101**, 4871–4876 (2004). 15041743 10.1073/pnas.0305627101 PMC387341

[20] Kato-Maeda, M., Bifani, P. J., Kreiswirth, B. N. & Small, P. M. The nature and consequence of genetic variability within *Mycobacterium tuberculosis*. *J. Clin. Invest.* **107**, 533–537 (2001). 11238552 10.1172/JCI11426 PMC199434

[21] Vargas, R. et al. Phase variation as a major mechanism of adaptation in *Mycobacterium tuberculosis* complex. *Proc. Natl. Acad. Sci. U. S. A.* **120**, e2301394120 (2023). 37399390 10.1073/pnas.2301394120 PMC10334774

[22] Cole, S. et al. Deciphering the biology of *Mycobacterium tuberculosis* from the complete genome sequence. *Nature* **396**, 190 (1998). 10.1038/31159 9634230

[23] Chen, B., Bajramović, B., Vriesendorp, B. & Spaink, H. P. Evolution of the PE_PGRS proteins of mycobacteria: Are all equal or are some more equal than others?. *Biology* **14**, 247 (2025). 40136504 10.3390/biology14030247 PMC11939664

[24] Kramarska, E., De Maio, F., Delogu, G. & Berisio, R. Structural basis of PE_PGRS polymorphism, a tool for functional modulation. *Biomolecules* **13**, 812 (2023). 37238682 10.3390/biom13050812 PMC10216338

[25] Machowski, E. E., Barichievy, S., Springer, B., Durbach, S. I. & Mizrahi, V. *In vitro* analysis of rates and spectra of mutations in a polymorphic region of the Rv0746 PE_PGRS gene of *Mycobacterium tuberculosis*. *J. Bacteriol.* **189**, 2190–2195 (2007). 17172340 10.1128/JB.01647-06 PMC1855714

[26] Talarico, S. et al. Variation of the *Mycobacterium tuberculosis* PE_PGRS33 gene among clinical isolates. *J. Clin. Microbiol.* **43**, 4954–4960 (2005). 16207947 10.1128/JCM.43.10.4954-4960.2005 PMC1248487

[27] Talarico, S. et al. *Mycobacterium tuberculosis* PE_PGRS16 and PE_PGRS26 genetic polymorphism among clinical isolates. *Tuberculosis* **88**, 283–294 (2008). 18313360 10.1016/j.tube.2008.01.001 PMC2562508

[28] Copin, R. et al. Sequence diversity in the PE_PGRS genes of *Mycobacterium tuberculosis* is independent of human T cell recognition. *MBio* **5**, e00960-e1013. 10.1128/mBio.00960-13 (2014). 24425732 10.1128/mBio.00960-13 PMC3903279

[29] Karboul, A. et al. Frequent homologous recombination events in *Mycobacterium tuberculosis* PE/PPE multigene families: Potential role in antigenic variability. *J. Bacteriol.* **190**, 7838–7846. 10.1128/JB.00827-08 (2008). 18820012 10.1128/JB.00827-08 PMC2583619

[30] Talarico, S. et al. *Mycobacterium tuberculosis* PE_PGRS16 and PE_PGRS26 genetic polymorphism among clinical isolates. *Tuberculosis (Edinb.)* **88**, 283–294 (2008). 18313360 10.1016/j.tube.2008.01.001 PMC2562508

[31] Hershberg, R. et al. High functional diversity in *Mycobacterium tuberculosis* driven by genetic drift and human demography. *PLoS Biol.* **6**, e311 (2008). 19090620 10.1371/journal.pbio.0060311 PMC2602723

[32] McEvoy, C. R. et al. Comparative analysis of *Mycobacterium tuberculosis* PE and PPE genes reveals high sequence variation and an apparent absence of selective constraints. *PLoS ONE* **7**, e30593. 10.1371/journal.pone.0030593 (2012). 22496726 10.1371/journal.pone.0030593 PMC3319526

[33] Zhang, Z., Dong, L., Li, X., Deng, T. & Wang, Q. The PE/PPE family proteins of *Mycobacterium tuberculosis*: Evolution, function, and prospects for tuberculosis control. *Front. Immunol.* **16**, 1606311. 10.3389/fimmu.2025.1606311 (2025). 40599786 10.3389/fimmu.2025.1606311 PMC12209268

[34] World Health Organization. Catalogue of mutations in Mycobacterium tuberculosis complex and their association with drug resistance. 2nd edn. Geneva: World Health Organization; 2023. Licence: CC BY-NC-SA 3.0 IGO. Available from: https://www.who.int/publications/i/item/9789240083295.

[35] Zuker, M. Mfold web server for nucleic acid folding and hybridization prediction. *Nucleic Acids Res.* **31**, 3406–3415 (2003). 12824337 10.1093/nar/gkg595 PMC169194

[36] Junier, I. Conserved patterns in bacterial genomes: A conundrum physically tailored by evolutionary tinkering. *Comput. Biol. Chem.* **53**, 125–133 (2014). 25239779 10.1016/j.compbiolchem.2014.08.017

[37] Noble, P. A., Citek, R. W. & Ogunseitan, O. A. Tetranucleotide frequencies in microbial genomes. *Electrophoresis* **19**, 528–535 (1998). 9588798 10.1002/elps.1150190412

[38] Lu, W., Zhang, Y., Liu, D., Songyang, Z. & Wan, M. Telomeres—Structure, function, and regulation. *Exp. Cell Res.* **319**, 133–141 (2013). 23006819 10.1016/j.yexcr.2012.09.005 PMC4051234

[39] Ratnatunga, C. N. et al. The rise of non-tuberculous mycobacterial lung disease. *Front. Immunol.* **11**, 303. 10.3389/fimmu.2020.00303 (2020). 32194556 10.3389/fimmu.2020.00303 PMC7062685

[40] Brazda, V., Fojta, M. & Bowater, R. P. Structures and stability of simple DNA repeats from bacteria. *Biochem. J.* **477**, 325–339. 10.1042/BCJ20190482 (2020). 31967649 10.1042/BCJ20190703 PMC7015867

[41] Bikard, D., Loot, C., Baharoglu, Z. & Mazel, D. Folded DNA in action: Hairpin formation and biological functions in prokaryotes. *Microbiol. Mol. Biol. Rev.* **74**, 570–588. 10.1128/MMBR.00026-10 (2010). 21119018 10.1128/MMBR.00026-10 PMC3008174

[42] Boyce, K. J. Mutators enhance adaptive micro-evolution in pathogenic microbes. *Microorganisms* **10**, 442 (2022). 35208897 10.3390/microorganisms10020442 PMC8875331

[43] Seligmann, H. & Pollock, D. D. The ambush hypothesis: Hidden stop codons prevent off-frame gene reading. *DNA Cell Biol.* **23**, 701–705 (2004). 15585128 10.1089/dna.2004.23.701

[44] Singh, P. K. et al. Mutations in SARS-CoV-2 leading to antigenic variations in spike protein: A challenge in vaccine development. *J. Lab. Physicians* **12**, 154–160 (2020). 32884216 10.1055/s-0040-1715790 PMC7462717

[45] Laha, S. et al. Characterizations of SARS-CoV-2 mutational profile, spike protein stability and viral transmission. *Infect. Genet. Evol.* **85**, 104445 (2020). 32615316 10.1016/j.meegid.2020.104445 PMC7324922

[46] Soczek, K. M., Grant, T., Rosenthal, P. B. & Mondragón, A. CryoEM structures of open dimers of gyrase A in complex with DNA illuminate mechanism of strand passage. *Elife* **7**, e41215. 10.7554/eLife.41215 (2018). 30457554 10.7554/eLife.41215 PMC6286129

[47] Wang, J. C. Cellular roles of DNA topoisomerases: A molecular perspective. *Nat. Rev. Mol. Cell Biol.* **3**, 430–440. 10.1038/nrm831 (2002). 12042765 10.1038/nrm831

[48] Mestre, O. et al. Phylogeny of *Mycobacterium tuberculosis* Beijing strains constructed from polymorphisms in genes involved in DNA replication, recombination and repair. *PLoS ONE* **6**, e16020. 10.1371/journal.pone.0016020 (2011). 21283803 10.1371/journal.pone.0016020 PMC3024326

[49] Kumar, A., Kamuju, V. & Vivekanandan, P. RNA G-quadruplexes inhibit translation of the PE/PPE transcripts in *Mycobacterium tuberculosis*. *J. Biol. Chem.* **300**, 105531. 10.1016/j.jbc.2023.105531 (2024). 38103641 10.1016/j.jbc.2023.105567 PMC10801317

[50] Snobre, J. et al. Frameshift mutations in the mmpR5 gene can have a bedaquiline-susceptible phenotype by retaining a protein structure and function similar to wild-type *Mycobacterium tuberculosis*. *Antimicrob. Agents Chemother.* **68**, e00854-e924. 10.1128/aac.00854-24 (2024). 39445816 10.1128/aac.00854-24 PMC11619236

