# Supplementary material for: Sequence-encoded determinants of regional mutational plasticity: comparative analysis of PE_PGRS genes in Mycobacterium tuberculosis and other bacteria
Source: Sci Rep. 2026 May 2;16:20400. doi: 10.1038/s41598-026-47170-w (PMC13328622; doi:10.1038/s41598-026-47170-w)
Supplement: Supplementary file 1 — Supplementary Information 1. [file 41598_2026_47170_MOESM1_ESM.docx]

**Suppl. Fig. S1.** A single-nucleotide insertion in the PE_PGRS 12 gene (Fig. S1A) of M. tuberculosis strain 5005 (CP049108.1) causes a frameshift mutation, leading to the loss of the canonical stop codon and elongation of the translated protein from 138 amino acids (Fig. S1B), as in M. tuberculosis H37Rv, to 380 amino acids (Fig. S1C). The newly formed stop codon is marked with an asterisk. Indels in gene PE_PGRS 12 in vaccine strain M. bovis BCG 35734 (CP109681.1) result in elongation of peptide to the full length.

**Suppl. Tab S1.** The table presents representative examples of predicted hairpin secondary structures encompassing mutation sites in bacterial, viral, and human genes. For each example, the mutation position, nucleotide change, predicted free energy (ΔG), melting temperature (Tm), and structural localization of the mutation are indicated

**Suppl. Tab. S2.** The thermodynamic comparison of hairpin-forming oligonucleotides with different stem compositions presented in Supplementary Table 1 demonstrates that the CGGC motif forms the most stable hairpin among all tested sequences, showing the lowest Gibbs free energy (ΔG = –4.33 kcal/mol) and the highest Tm values, followed by CGGG, CGCG, GGG, GGGG, and the non-GC-rich controls. These stronger interactions in the CGGC stem potentially enable more pronounced energy redistribution during replication, which may lead to nucleotide misincorporation. These observations support a model in which mutagenesis is driven by structurally constrained, physiochemically mediated interactions rather than by stochastic errors alone.

**Suppl. Tab. S3.** Data in Supplementary Table S3 compare the abundance of CGGC tetramers and out-of-frame pre-stop codons controlling 1-nt and 2-nt frameshift robustness with the frequency of mutator strains ^32^ in bacterial populations. Species with higher mutator frequencies tend to have increased CGGC content and reduced pre-stop codon levels, such as *Pseudomonas aeruginosa* (19.5–92%)^32^, and *Neisseria meningitidis* (22.2–56.8%)^32^. In contrast, species with low mutator rates, including *Staphylococcus aureus* (1.4–14.6%)^32,^ and *Vibrio parahaemolyticus* (0–5.1%)^32^, display lower CGGC levels and higher pre-stop codon abundance

**Suppl. Tab. S4.** List of 88 bacterial genomes, (74 species) included in the study, with corresponding GenBank accession numbers, (NCBI)
