# Supplementary material for: Sequence-encoded determinants of regional mutational plasticity: comparative analysis of PE_PGRS genes in Mycobacterium tuberculosis and other bacteria
Source: Sci Rep. 2026 May 2;16:20400. doi: 10.1038/s41598-026-47170-w (PMC13328622; doi:10.1038/s41598-026-47170-w)
Supplement: Supplementary file 2 — Supplementary Information 2. [file 41598_2026_47170_MOESM2_ESM.docx]

| **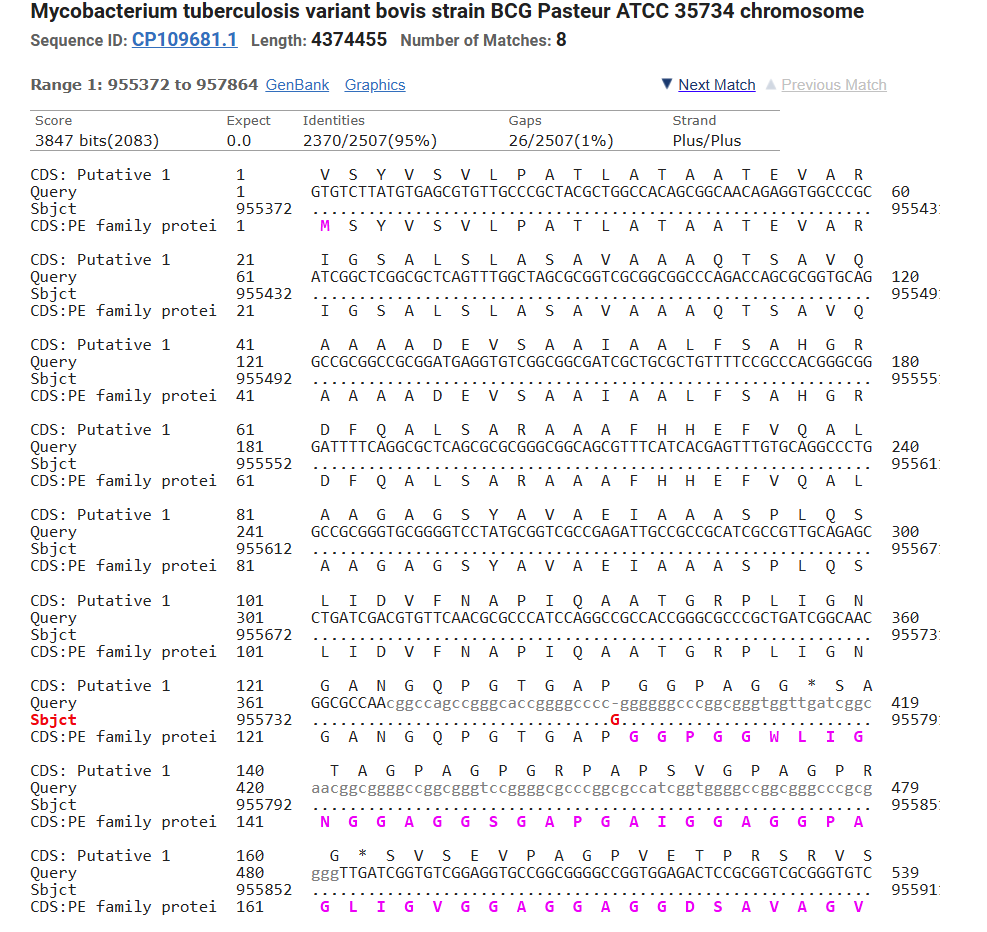**  **А**  (A) Indel in Mtb strain 5005 leads to extension of the open reading frame.  (B) The translated product in Mtb H37Rv consists of 138 amino acids.  (C) The translated product in strain Mtb 5005 consists of 380 amino acids. | **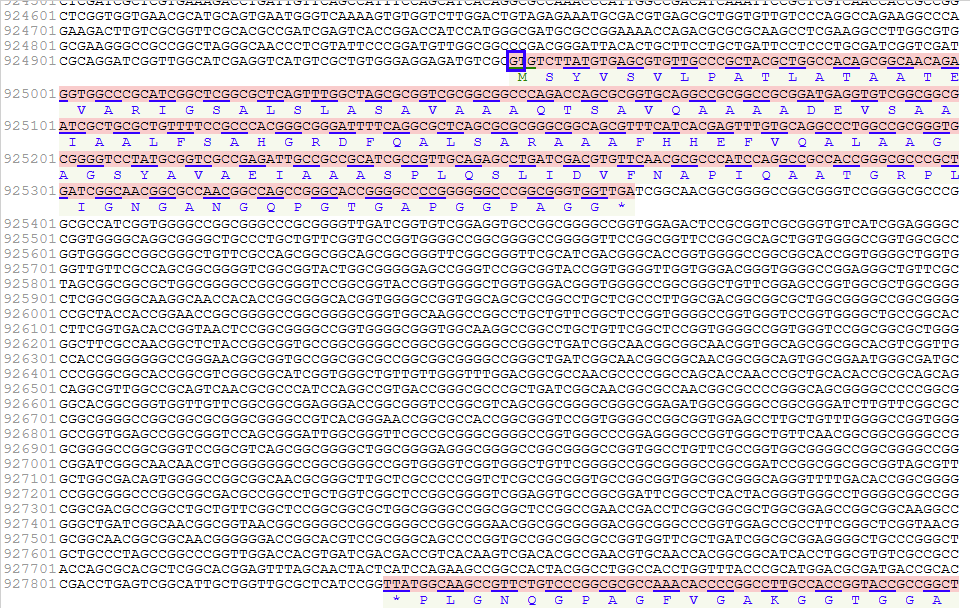**  **В** |
| --- | --- |
|  | **­­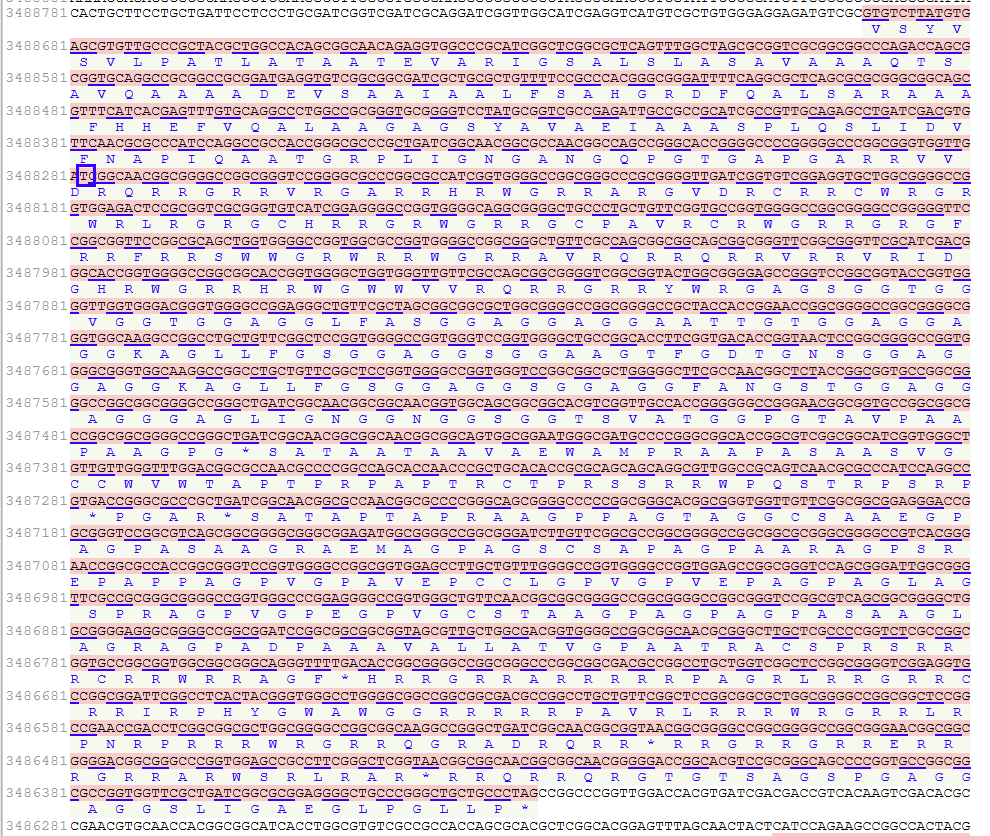**  **С** |

**Suppl. Fig. S1.** **Indel-driven frame shift mutation and peptide elongation in the PE_PGRS 12 gene in M. tuberculosis strain 5005 (CP049108.1) versus M. tuberculosis H37Rv**
