# Supplementary material for: Sequence-encoded determinants of regional mutational plasticity: comparative analysis of PE_PGRS genes in Mycobacterium tuberculosis and other bacteria
Source: Sci Rep. 2026 May 2;16:20400. doi: 10.1038/s41598-026-47170-w (PMC13328622; doi:10.1038/s41598-026-47170-w)
Supplement: Supplementary file 3 — Supplementary Information 3. [file 41598_2026_47170_MOESM3_ESM.docx]

**Supplementary Table S1 |** Representative hairpin secondary structures at mutation sites across different genes and taxa

| Gene | Genome | | | | | Mutation | | | Hairpin secondary structures |
| --- | --- | --- | --- | --- | --- | --- | --- | --- | --- |
| Hairpin secondary structures at mutation sites in PE_PGRS genes | | | | | | | | | |
| PE_PGRS7 | Mtb H37Rv vs 4860 (CP049108.1) | | | | | G302A | | | 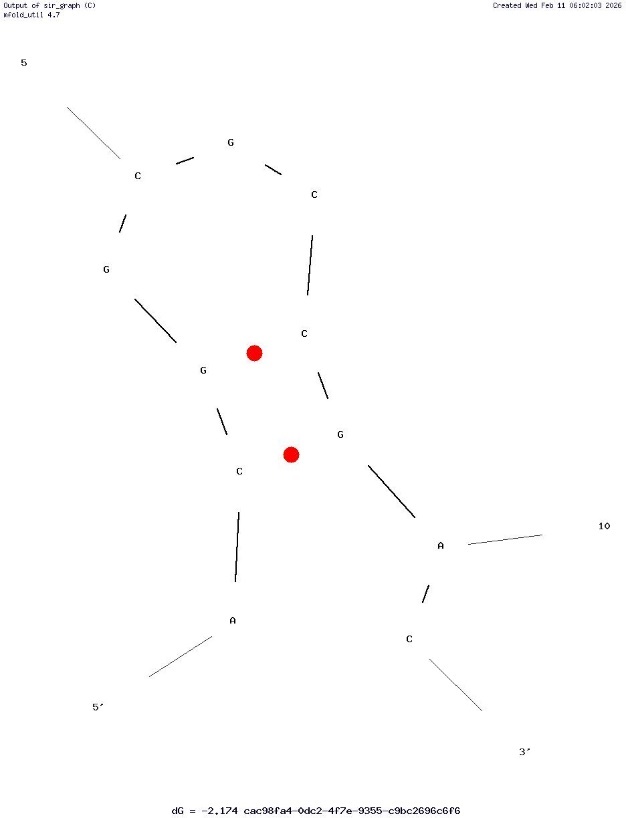 |
|  | ΔG (kcal/mol) | | ΔH  (kcal/mol) | | | ΔS (cal/mol·K) | Tm (°C) | |  |
|  | -2.17 | | 52.2 | | | -26 | -79.91 | |  |
|  | Sequence (5'→3') | | | | | | | |  |
|  | acggc[G]ccgac | | | | | | | |  |
| PE_PGRS7 | Mtb H37Rv vs 4860 (CP053092.1) | | | | | C4T | | | A  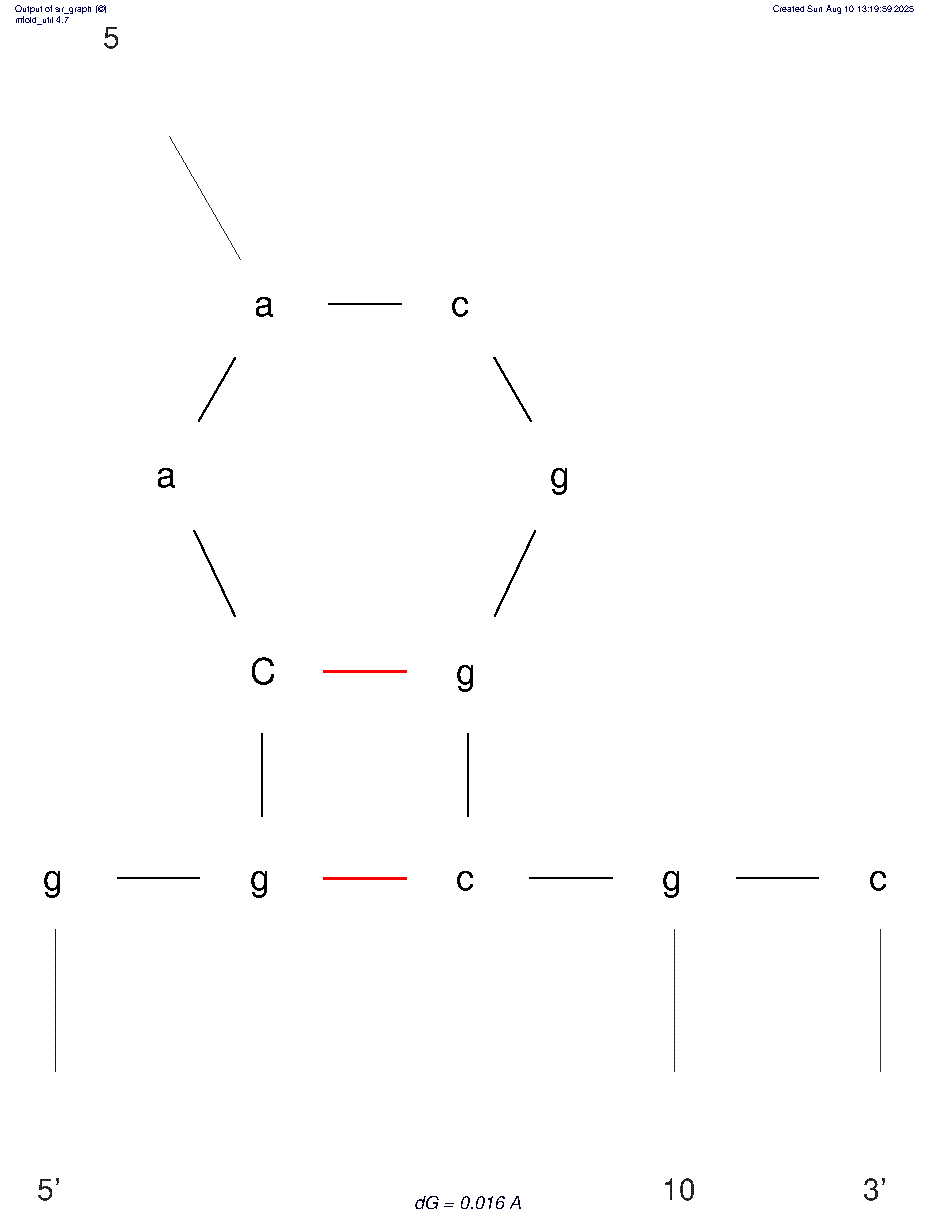  B  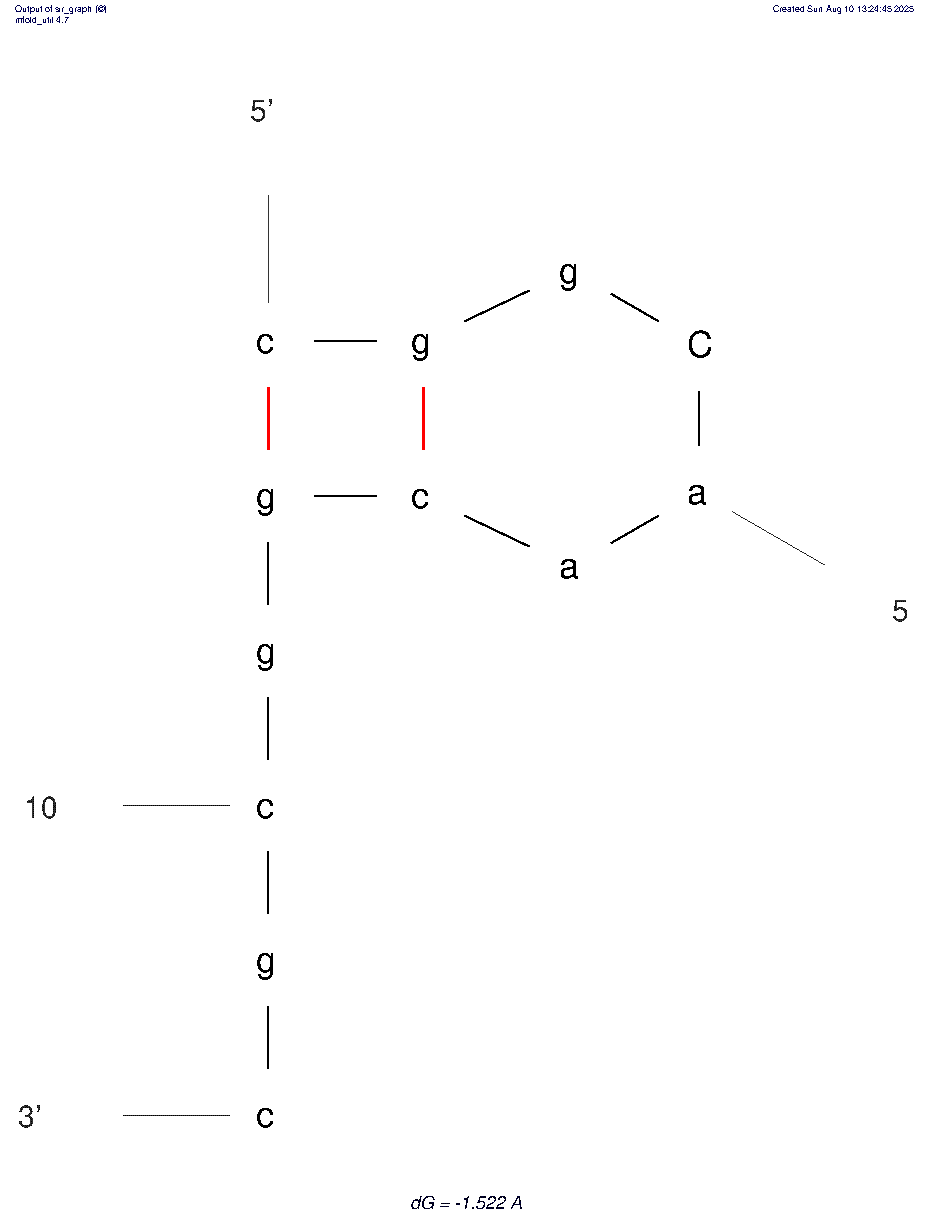 |
|  | ΔG (kcal/mol) | | ΔH  (kcal/mol) | | | ΔS (cal/mol·K) | Tm (°C) | |  |
|  | A - 0.01 | | 16.70 | | | -53.81 | 37.2°C | |  |
|  | B -1.52 | | - 20.10 | | | -59.91 | 62.4°C | |  |
|  | Sequence (5'→3') | | | | | | | |  |
|  | ccgg[C]aacggcgccgacggggcggccggg | | | | | | | |  |
| PE_PGRS7 | Mtb H37Rv vs 4860 (CP053092.1) | | | | | A14T | | | 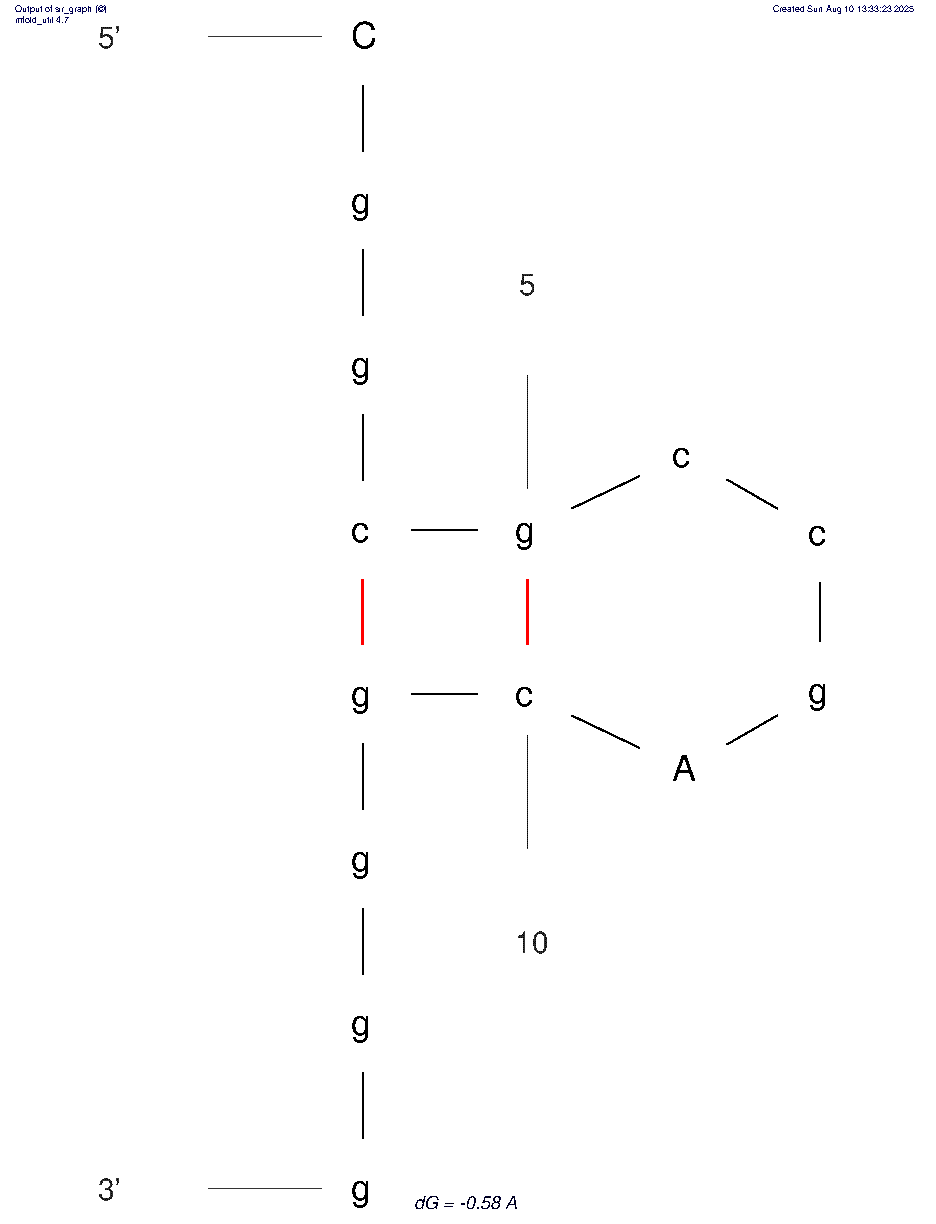 |
|  | ΔG (kcal/mol) | | ΔH  (kcal/mol) | | | ΔS (cal/mol·K) | Tm (°C) | |  |
|  | -0.58 | | -22.40 | | | -70.35 | 45.3°C | |  |
|  | Sequence (5'→3') | | | | | | | |  |
|  | Cggcgccg[A]cgggg | | | | | | | |  |
| PE_PGRS7 | H37Rv vs Mtb 4860 (CP053092.1) | | | | | G45C | | | 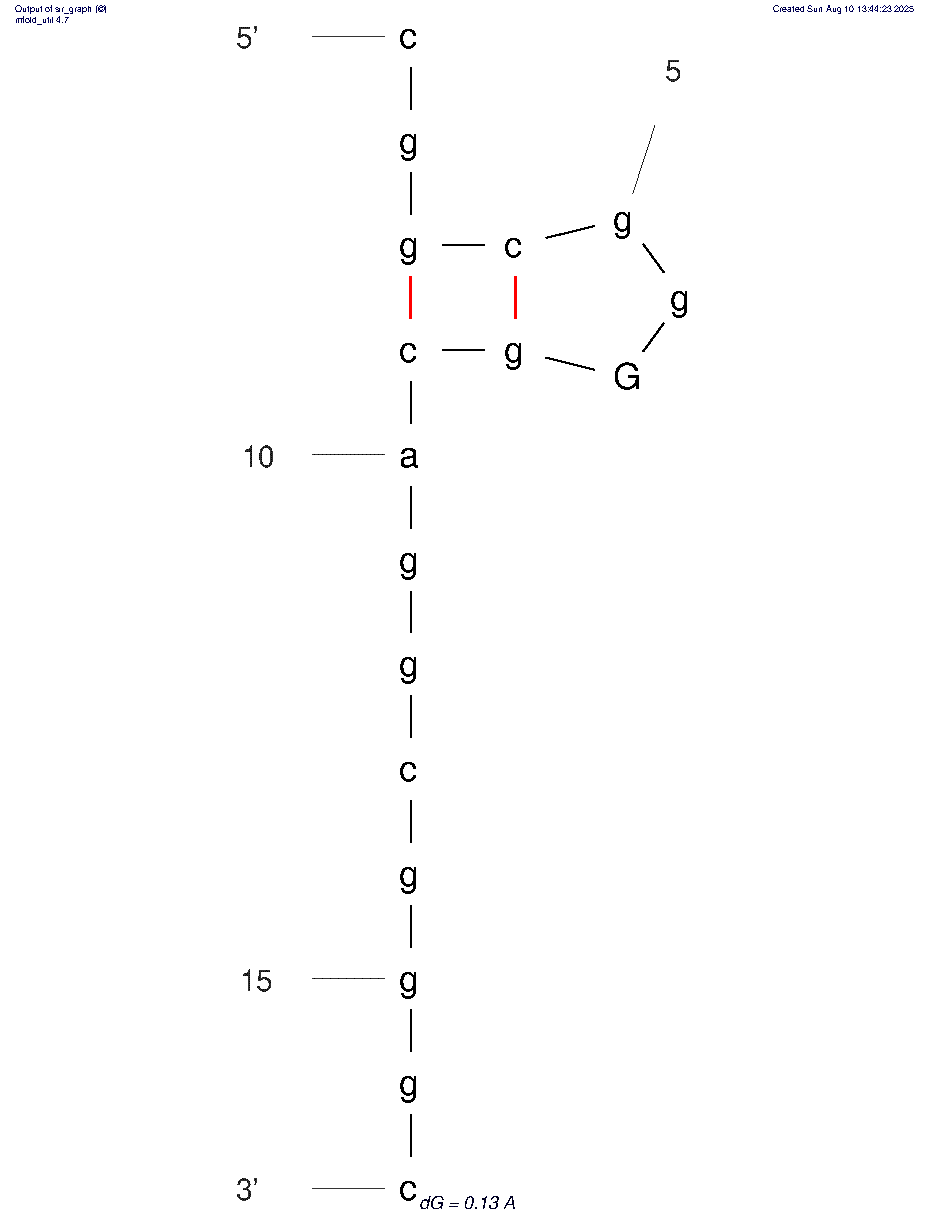 |
|  | ΔG (kcal/mol) | | ΔH  (kcal/mol) | | | ΔS (cal/mol·K) | Tm (°C) | |  |
|  | +0.13 | | -19.60 | | | -63.61 | 35.0°C | |  |
|  | Sequence (5'→3') | | | | | | | |  |
|  | cggcgg[G]gcaggcgggc | | | | | | | |  |
| PE_PGRS7 | H37Rv vs Mtb 4860 (CP053092.1) | | | | | A48G  G50A | | | 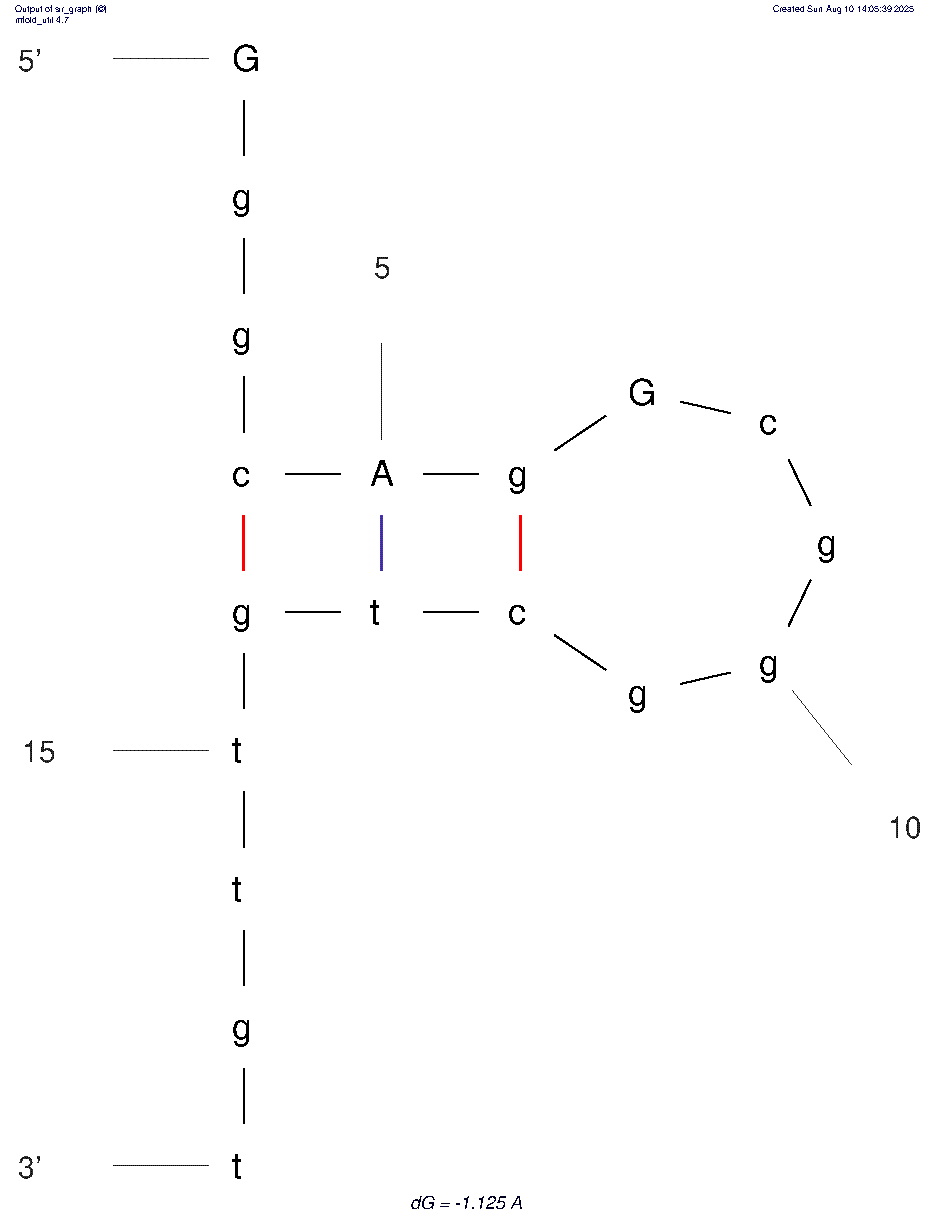 |
|  | ΔG (kcal/mol) | | ΔH  (kcal/mol) | | | ΔS (cal/mol·K) | Tm (°C) | |  |
|  | -1.12 | | -31.10 | | | -96.66 | 48.6°C | |  |
|  | Sequence (5'→3') | | | | | | | |  |
|  | Gggc[A]g[G]cgggctgttgt | | | | | | | |  |
| PE_PGRS 53 | H37Rv vs *M. bovis* BCG Pasteur ATCC 35734  CP109681.1 | | | | | G505A  G506T | | | 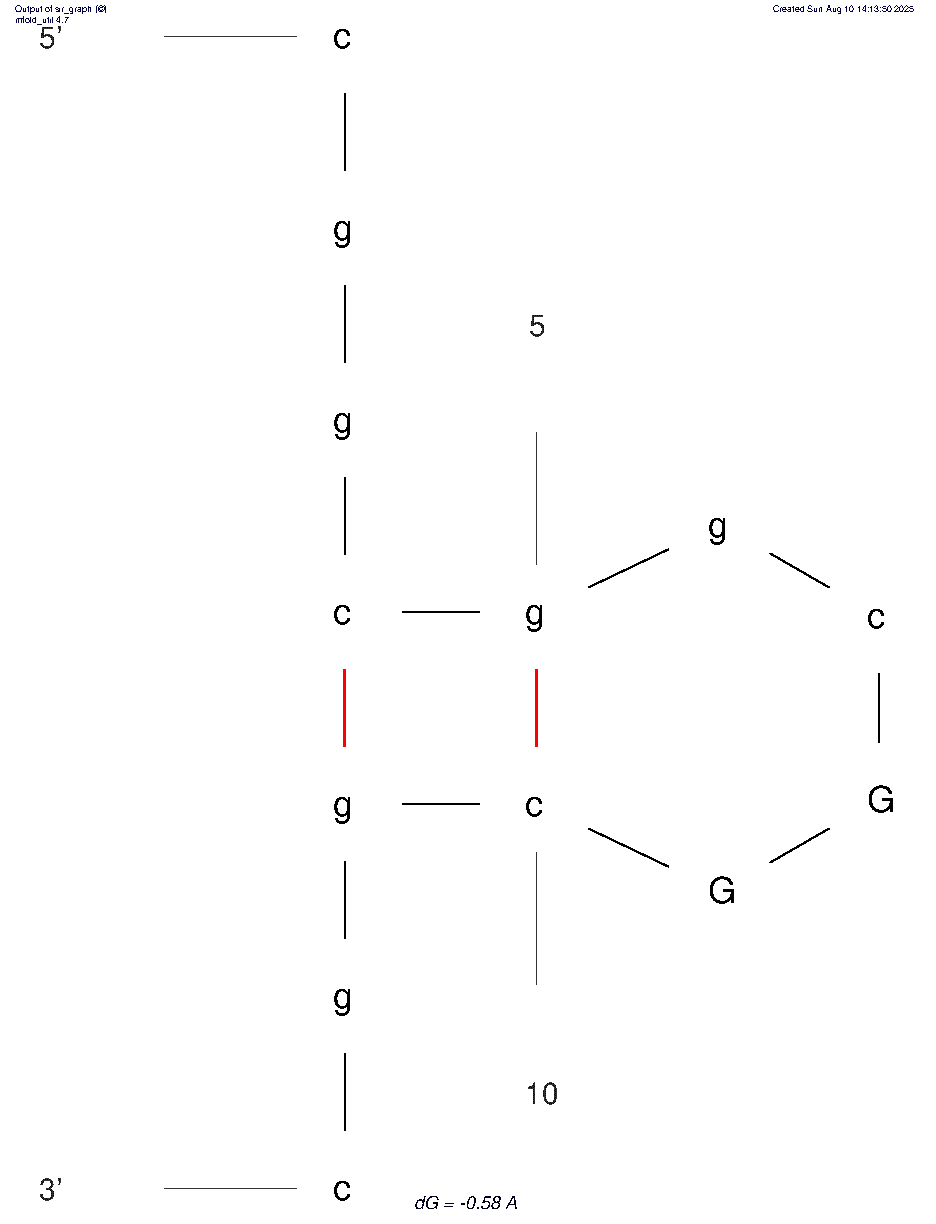 |
|  | ΔG (kcal/mol) | | ΔH  (kcal/mol) | | | ΔS (cal/mol·K) | Tm (°C) | |  |
|  | -0.58 | | -24.90 | | | -78.41 | 44.4°C | |  |
|  | Sequence (5'→3') | | | | | | | |  |
|  | Ccggcggc[GG]cggcggggccggcgc  cggcggc[GG]cggc | | | | | | | |  |
| PE_PGRS 53 | | Mtb H37Rv vs M*. bovis* BCG Pasteur _ATCC 35734_  CP109681.1 | | | C210G, G213C, C216G | | | | 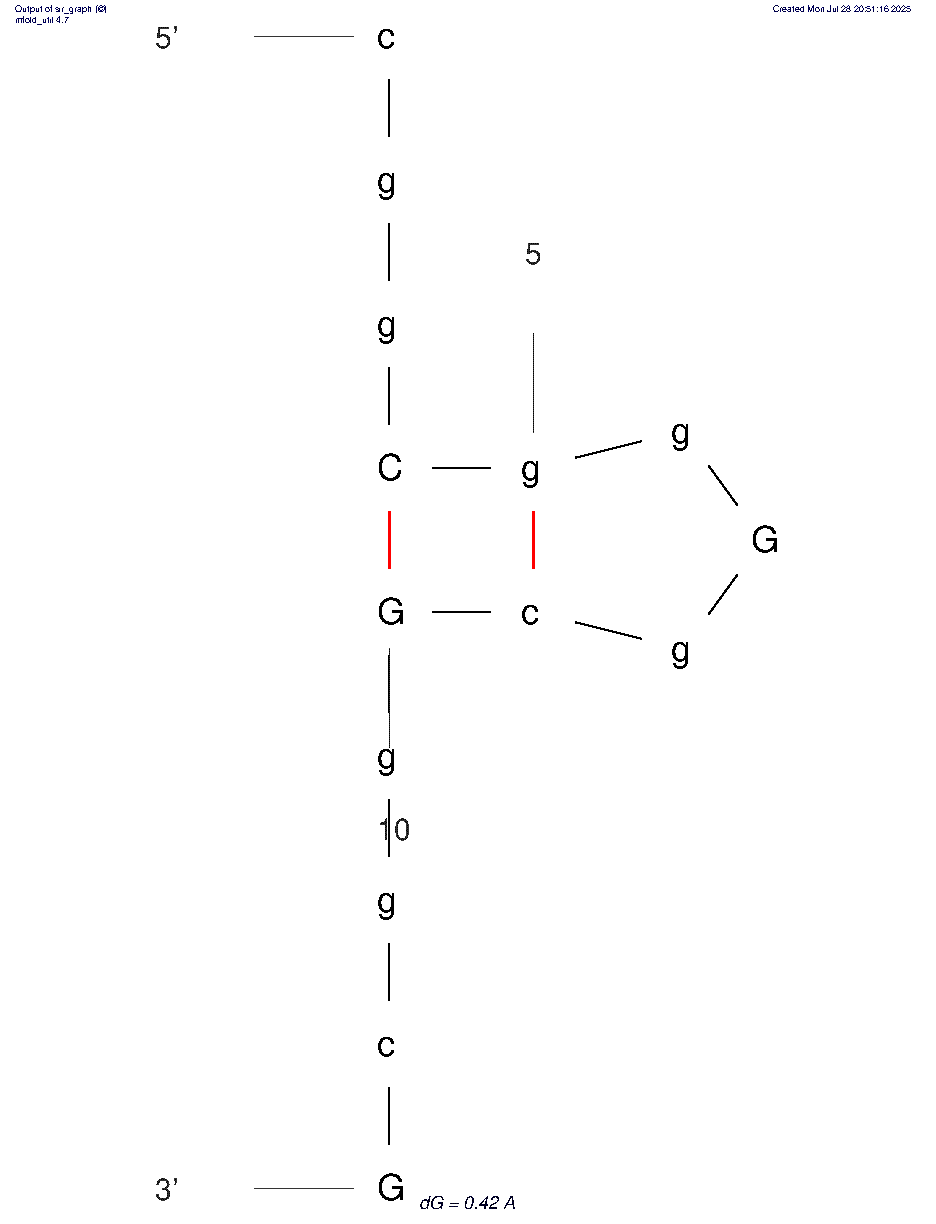 |
|  |  | ΔG (kcal/mol) | | ΔH  (kcal/mol) | ΔS (cal/mol·K) | | | Tm (°C) |  |
|  |  | +0.42 | | -19.60 | -64.55 | | | 30.5°C |  |
|  |  | Sequence (5'→3') | | | | | | |  |
|  |  | cgg[C]gg[G]gc[G]ggc[G]c | | | | | | |  |
| PE_PGRS 53 | | H37Rv vs *M. bovis* BCG Pasteur ATCC 35734  CP109681.1 | | | C213G  G217A | | | | 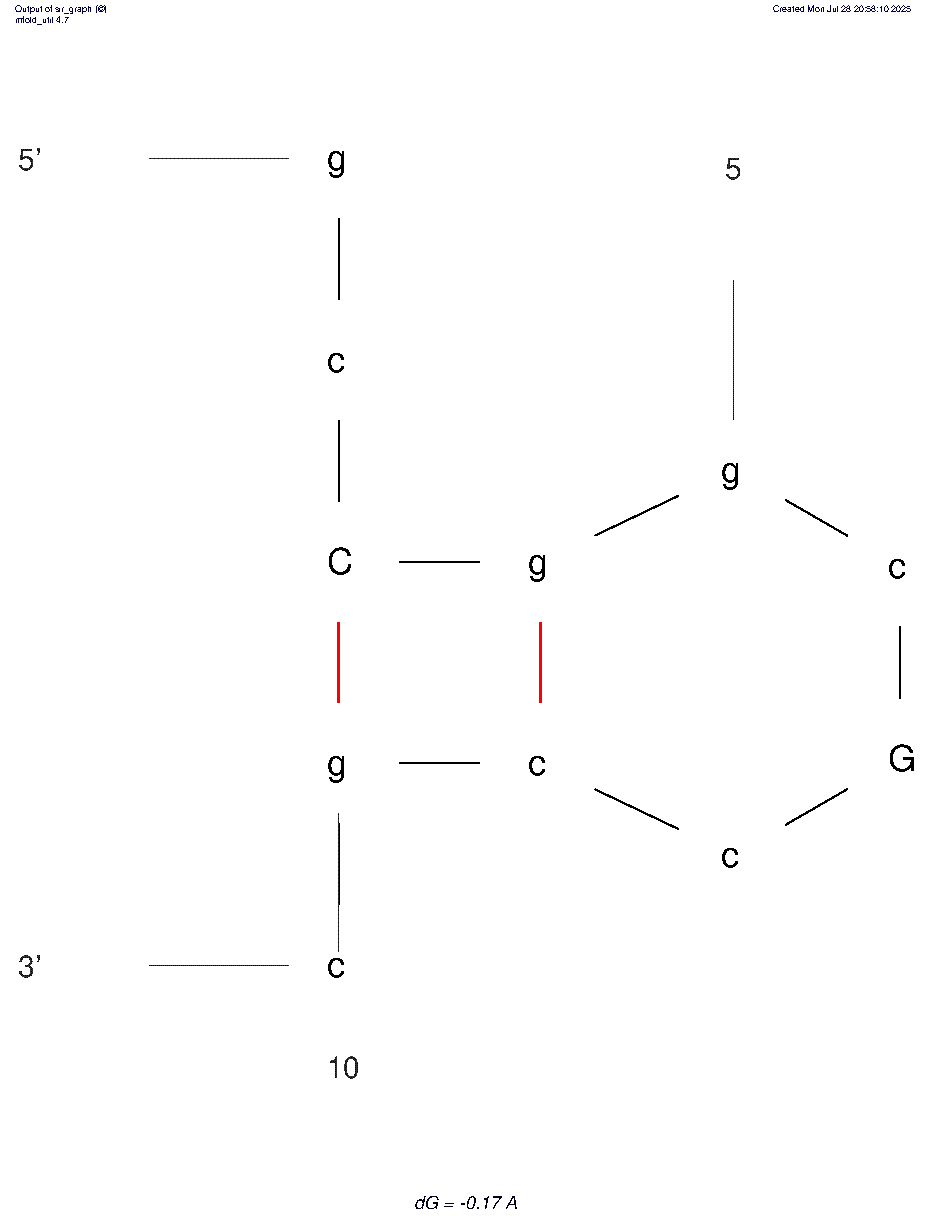 |
|  |  | ΔG (kcal/mol) | | ΔH  (kcal/mol) | ΔS (cal/mol·K) | | | Tm (°C) |  |
|  |  | -0.17 | | -22.20 | -71.03 | | | 39.4°C |  |
|  |  | Sequence (5'→3') | | | | | | |  |
|  |  | gc[C]ggc[G]ccgc | | | | | | |  |
| Hairpin secondary structures at mutation sites in ligD, ligB | | | | | | | | | |
| ligD | | Mtb H37Rv vs 4860 (CP053092.1) | | | TGC1030CGC | | | | 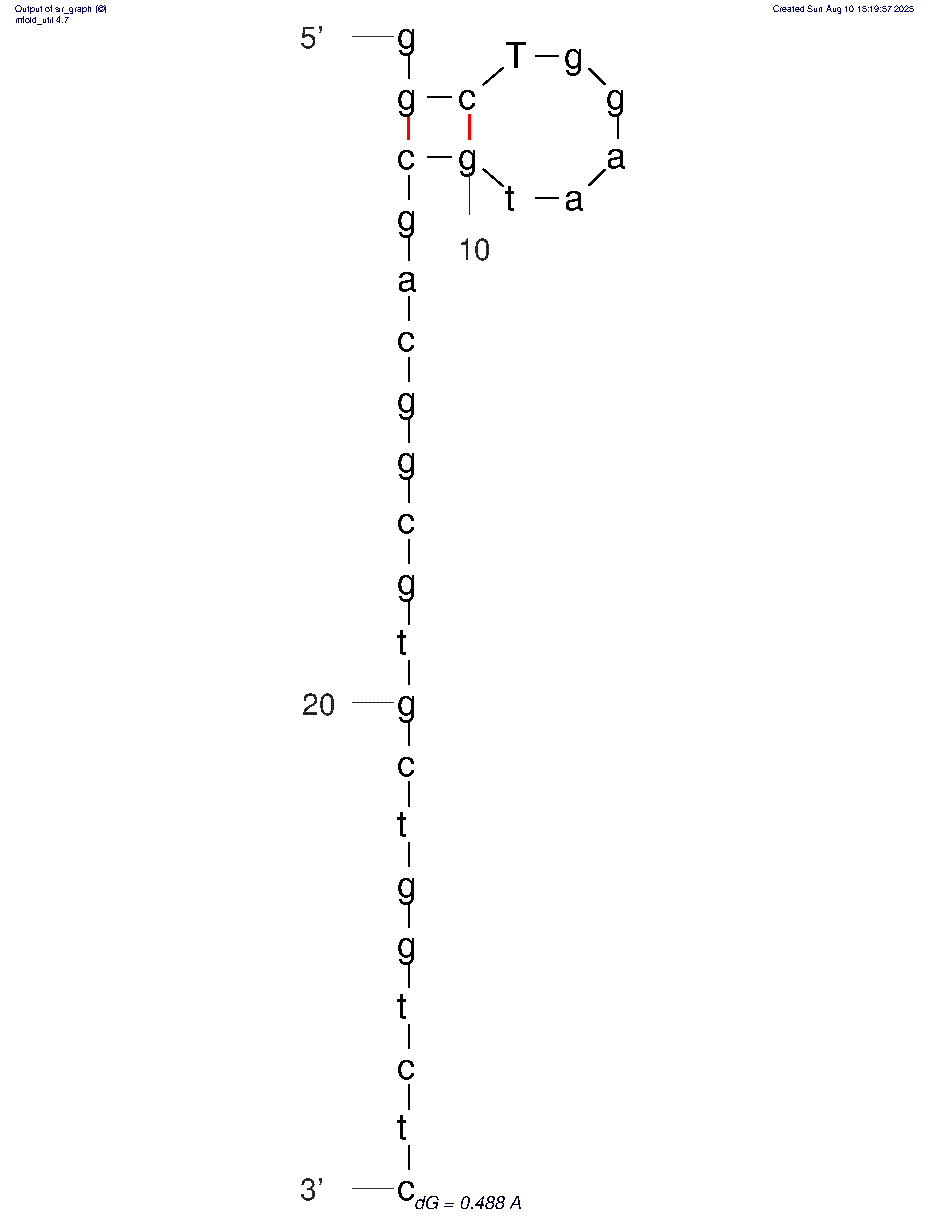 |
|  |  | ΔG (kcal/mol) | | ΔH  (kcal/mol) | ΔS (cal/mol·K) | | | Tm (°C) |  |
|  |  | -0.50 | | -18.50 | -58.04 | | | 45.6°C |  |
|  |  | Sequence (5'→3') | | | | | | |  |
|  |  | Сggc[T]ggaatgcgacggcgtgctggtctc | | | | | | |  |
| ligB | | Mtb H37Rv vs 4860 (CP053092.1) | | | CCG271TCG | | | | 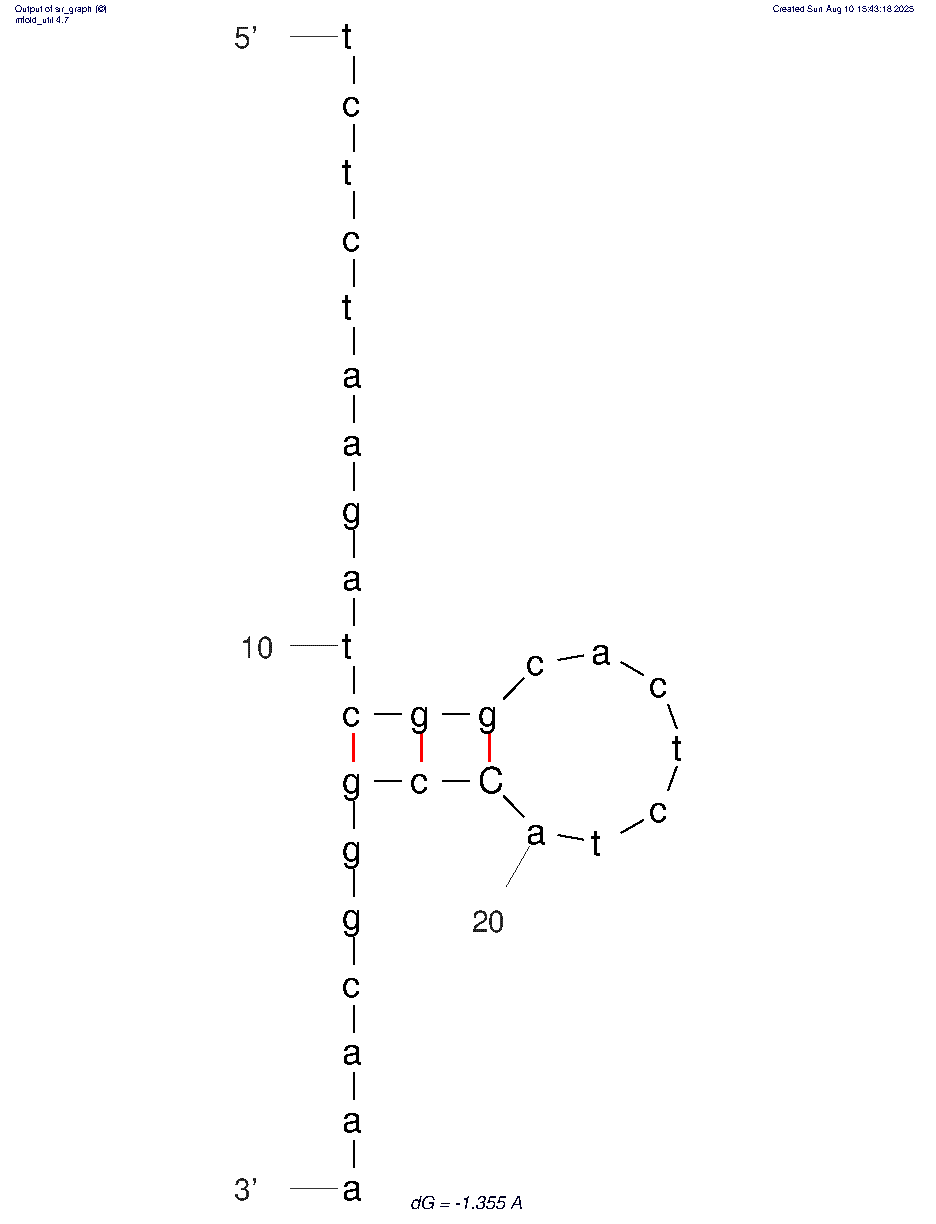 |
|  |  | ΔG (kcal/mol) | | ΔH  (kcal/mol) | ΔS (cal/mol·K) | | | Tm (°C) |  |
|  |  | -0.50 | | -18.50 | -58.04 | | | 45.6°C |  |
|  |  | Sequence (5'→3') | | | | | | |  |
|  |  | tctctaagatcggcactcta[C]cgggcaaa | | | | | | |  |
| Hairpin secondary structures at mutation sites in Human immunodeficiency virus type 1 (HXB2), HIV1/HTLV-III/LAV; GenBank: K03455.1 | | | | | | | | | |
| pol | | HIV1/HTLV-III/LAV;  GenBank: K03455.1 | | | **M184V**  ATG→GTG | | | | A  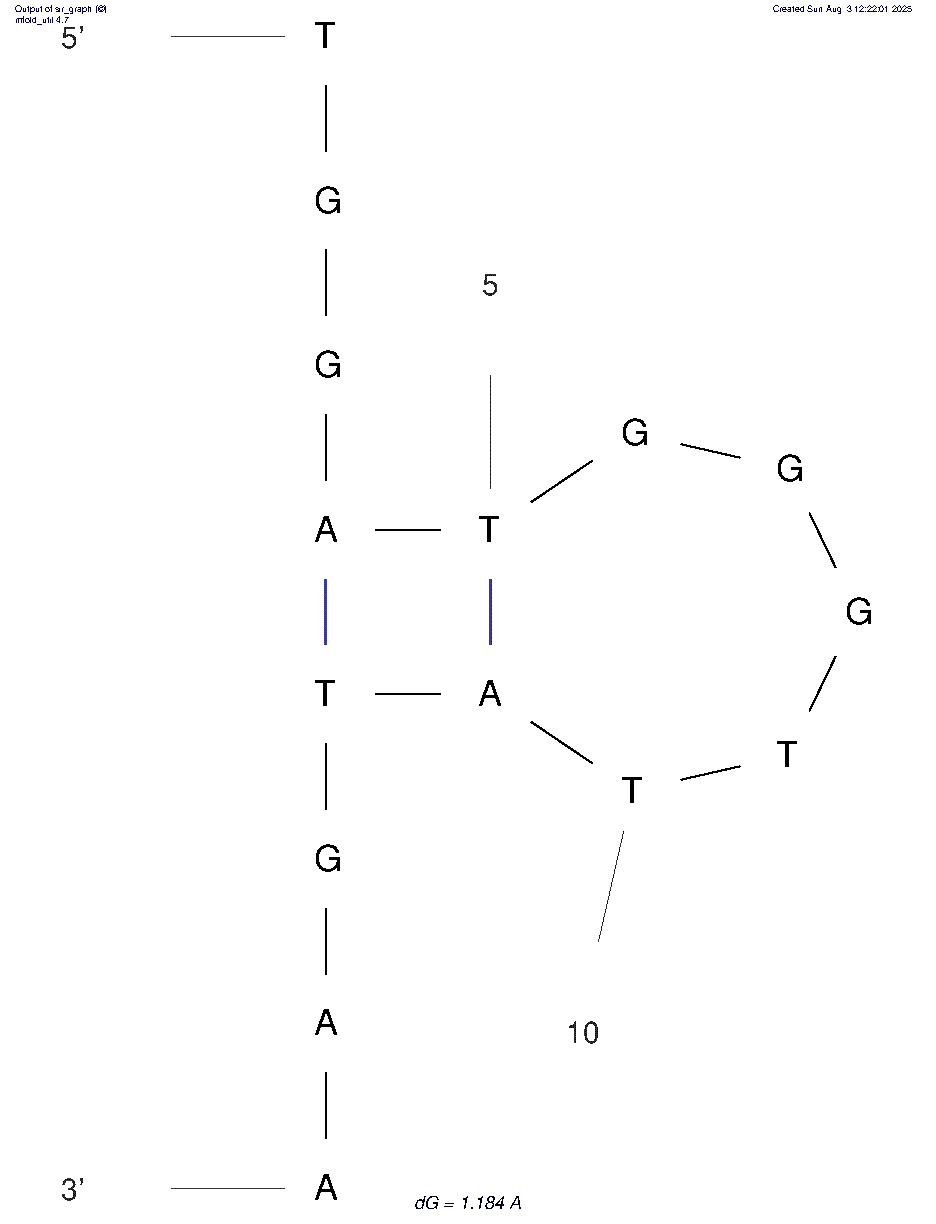  B  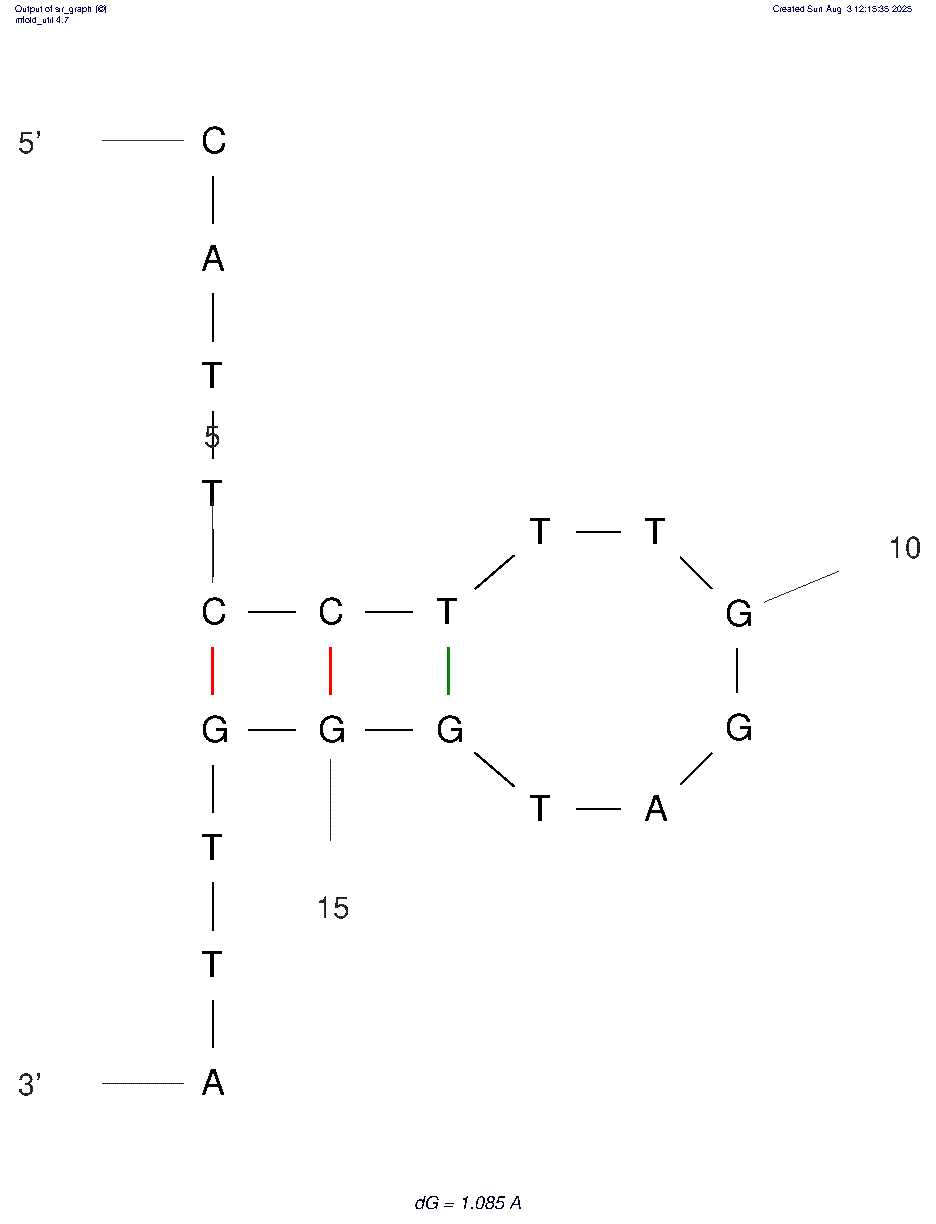 |
|  |  | ΔG (kcal/mol) | | ΔH  (kcal/mol) | ΔS (cal/mol·K) | | | Tm (°C) |  |
|  |  | A +1.18 | | -10.00 | -36.05 | | | 4.2°C |  |
|  |  | B +1.09 | | -19.20 | -65.42 | | | 20.3°C |  |
|  |  | Sequence (5'→3') | | | | | | |  |
|  |  | cattcctttgg[A]tgggttatgaactccatcctgataaa  [A]tgggttatgaactccatcctgataaa | | | | | | |  |
| Hairpin secondary structures at mutation sites in SARS-CoV-2 (Gene ID: 43740568, NC_045512.2 (21563..25384)^44, 45^ | | | | | | | | | |
| S- Spike Protein | | SARS-CoV-2 (Gene ID: 43740568, NC_045512.2 (21563..25384) | | | D614 ^44, 45^  A→G | | | | A  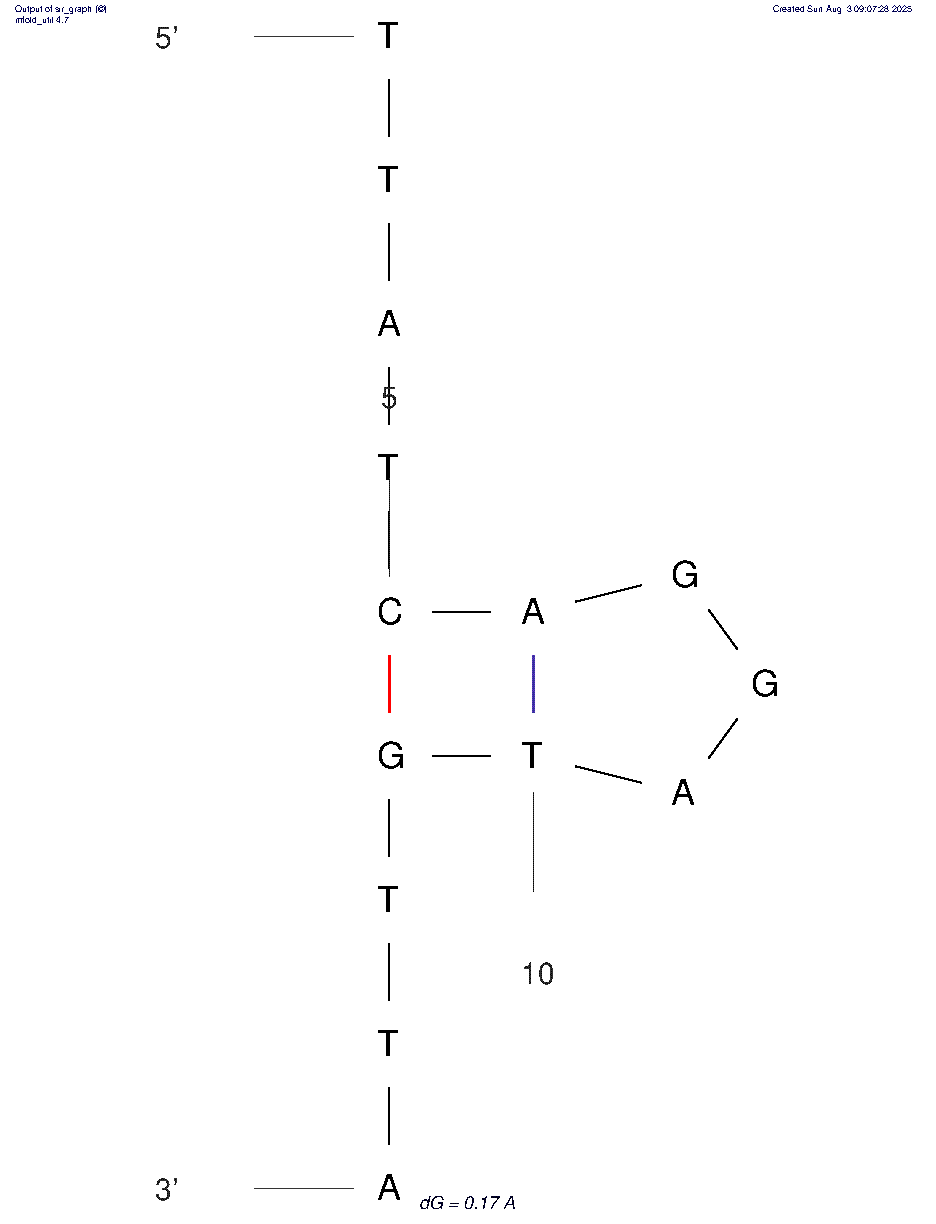  B  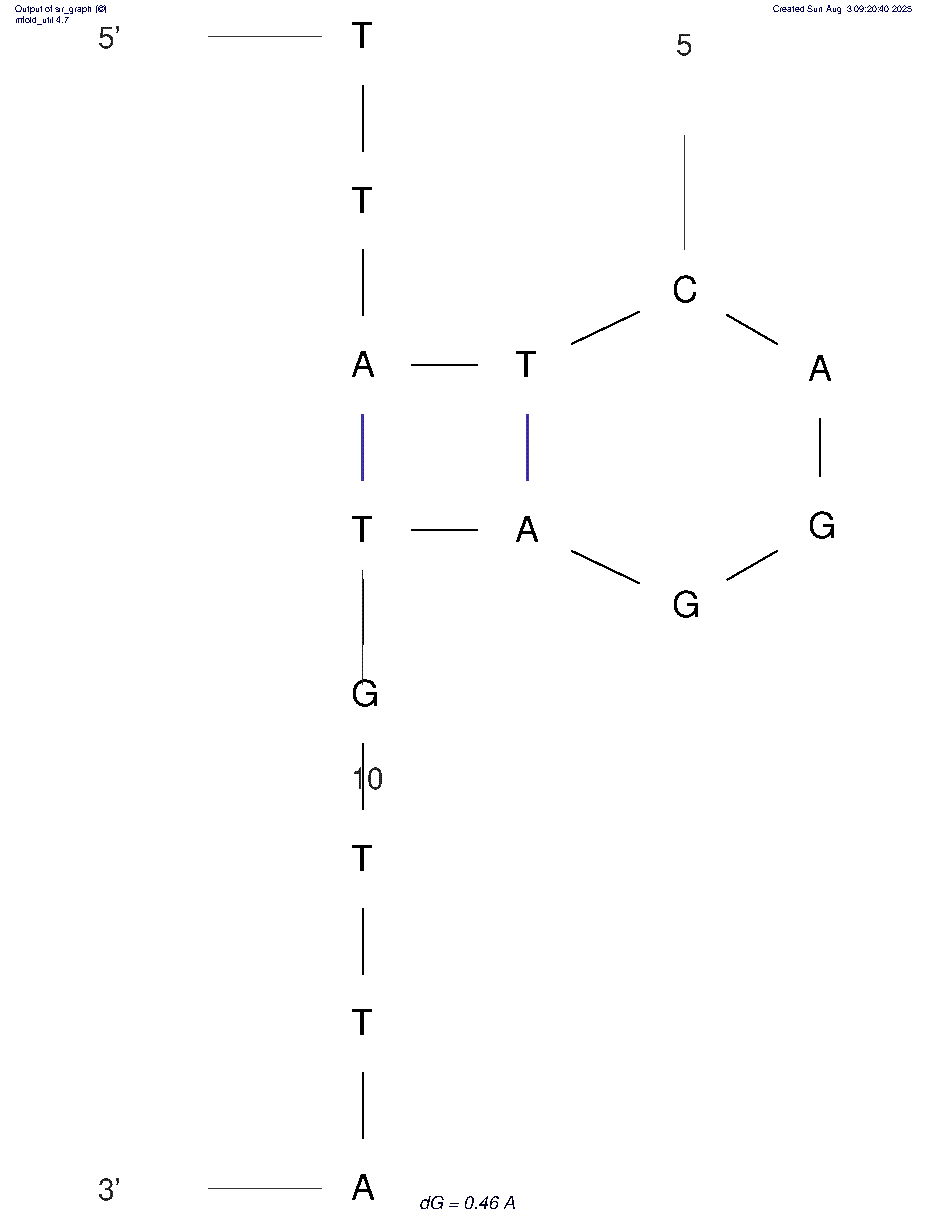 |
|  |  | ΔG (kcal/mol) | | ΔH  (kcal/mol) | ΔS (cal/mol·K) | | | Tm (°C) |  |
|  |  | A +0.17 | | -15.70 | -51.17 | | | 33.7°C |  |
|  |  | B +0.46 | | -19.50 | = -64.36 | | | 29.8°C |  |
|  |  | Sequence (5'→3') | | | | | | |  |
|  |  | cagg[A]tgtt | | | | | | |  |
| S- Spike Protein | | SARS-CoV-2 (Gene ID: 43740568, NC_045512.2 (21563..25384) | | | 292 ALDP 295^44, 45^ | | | | A  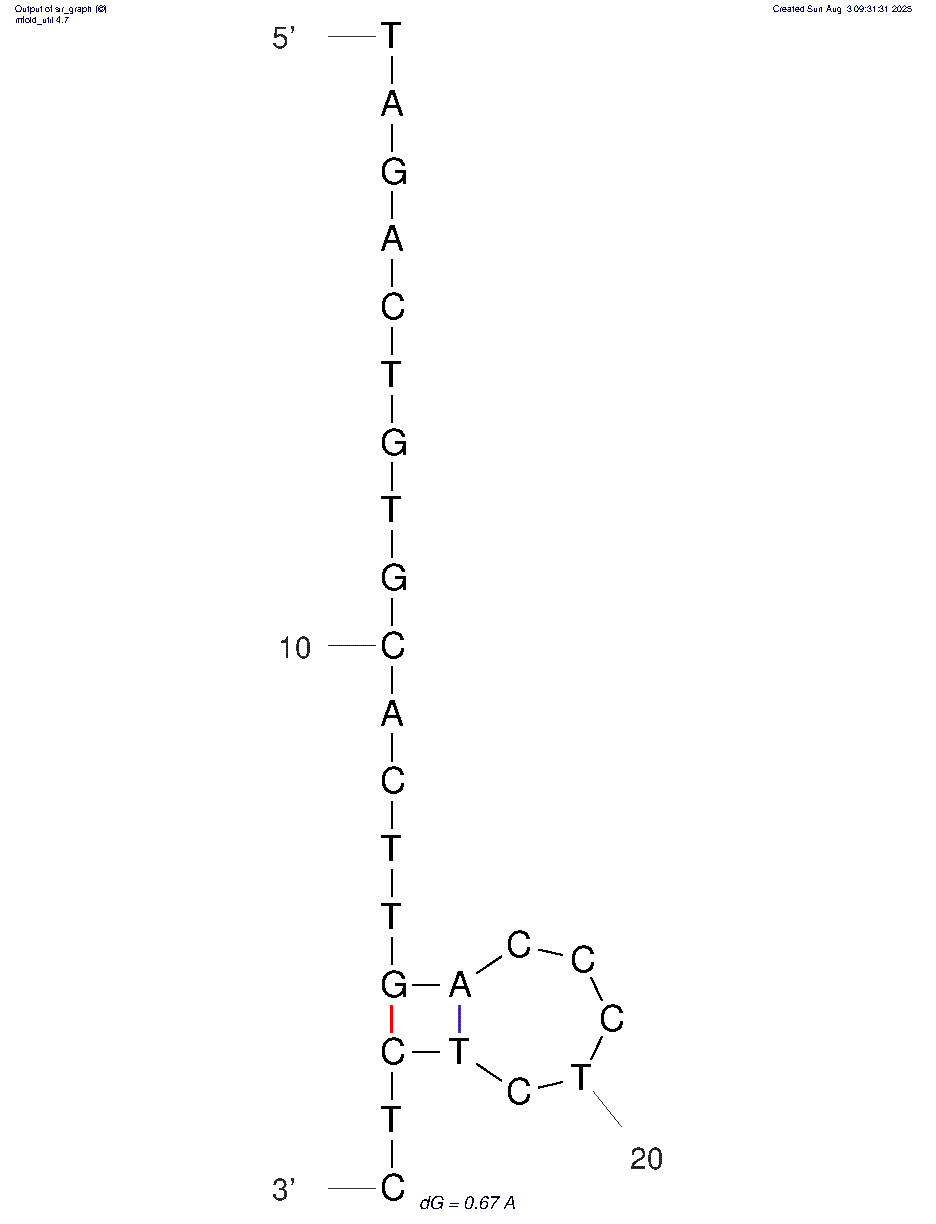  B  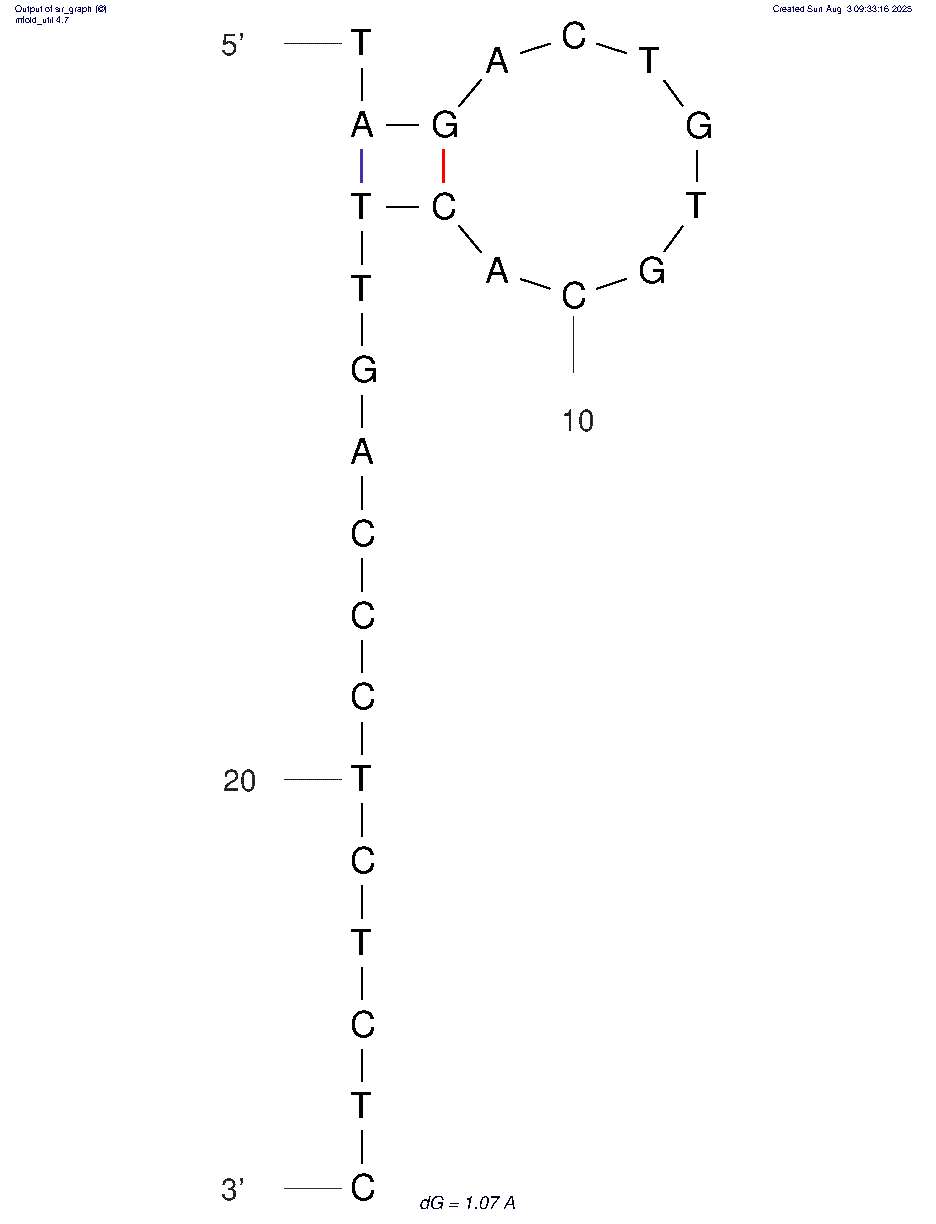 |
|  |  | ΔG (kcal/mol) | | ΔH  (kcal/mol) | ΔS (cal/mol·K) | | | Tm (°C) |  |
|  |  | A +0.67 | | -18.80 | -62.78 | | | 26.3°C |  |
|  |  | B +1.07 | | -15.60 | -53.75 | | | 17.1°C |  |
|  |  | Sequence (5'→3') | | | | | | |  |
|  |  | gCACTTGACCct | | | | | | |  |
| S- Spike Protein | | SARS-CoV-2 (Gene ID: 43740568, NC_045512.2 (21563..25384) | | | L54W  Ttg→tgg^44, 45^ | | | | 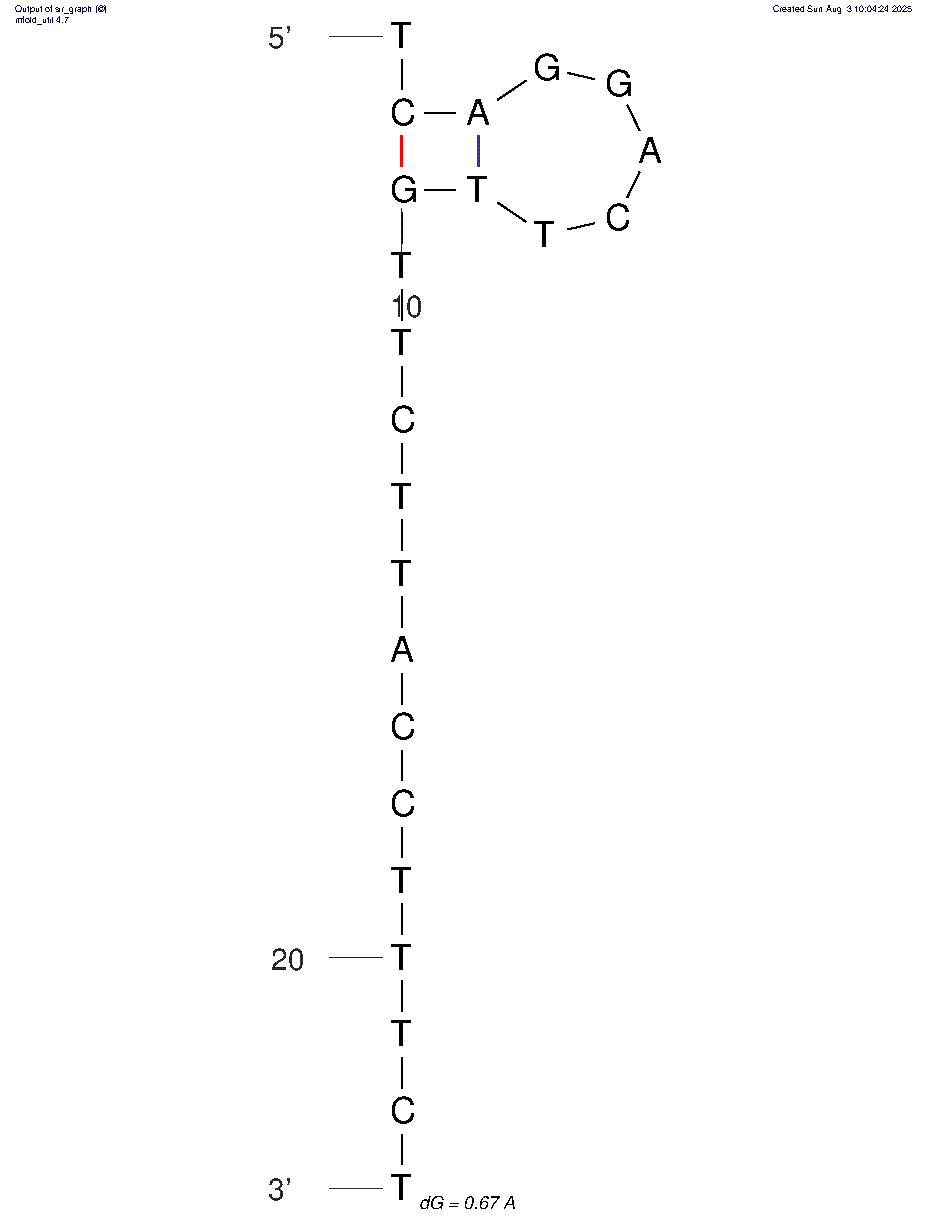 |
|  |  | ΔG (kcal/mol) | | ΔH  (kcal/mol) | ΔS (cal/mol·K) | | | Tm (°C) |  |
|  |  | -0.00 | | -16.10 | -51.91 | | | 37.0°C |  |
|  |  | Sequence (5'→3') | | | | | | |  |
|  |  | gact[T]g[T]tctta | | | | | | |  |
| S- Spike Protein | | SARS-CoV-2 (Gene ID: 43740568, NC_045512.2 (21563..25384) | | | F55I  Ttc→atc ^44, 45^ | | | | 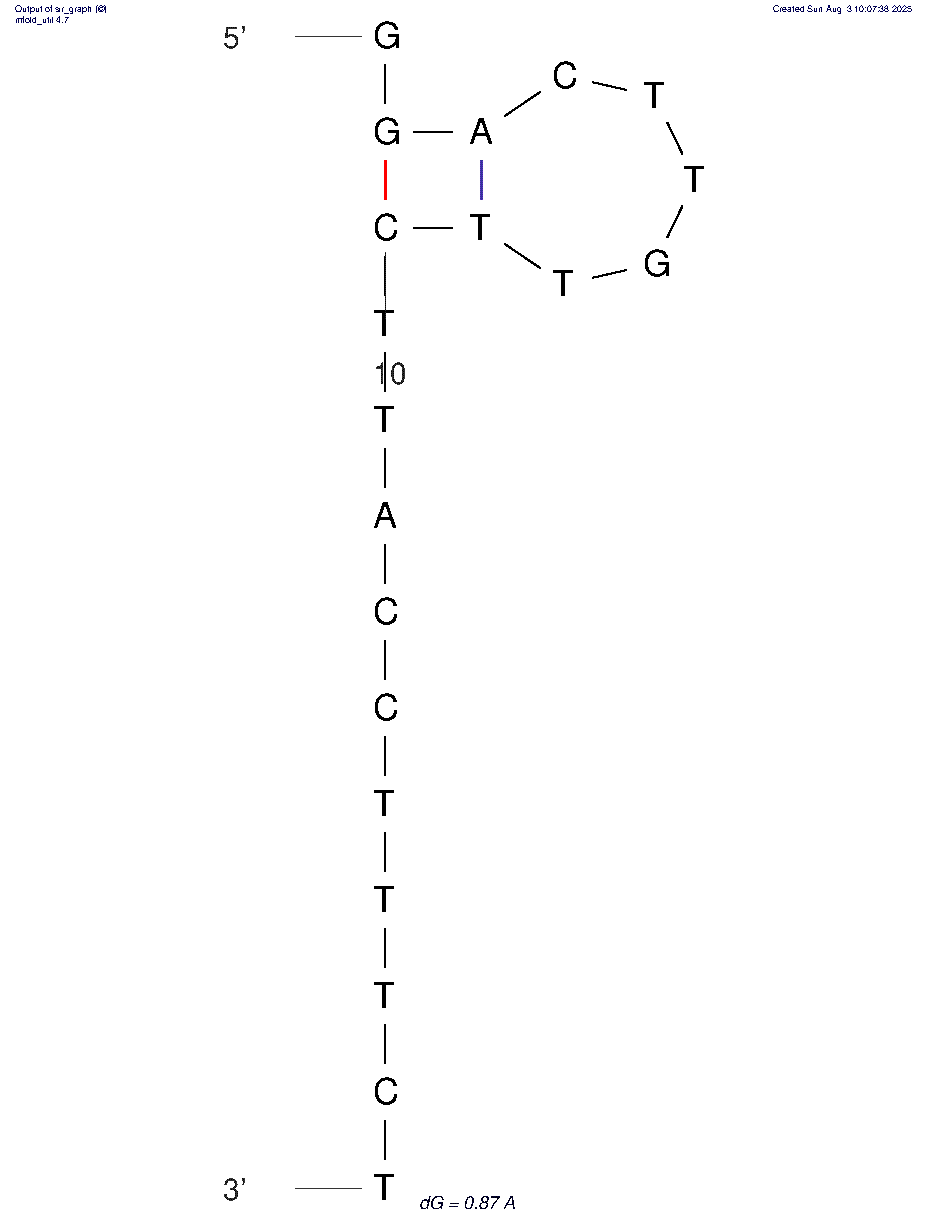 |
|  |  | ΔG (kcal/mol) | | ΔH  (kcal/mol) | ΔS (cal/mol·K) | | | Tm (°C) |  |
|  |  | +0.87 | | -18.50 | -62.45 | | | 23.1°C |  |
|  |  | Sequence (5'→3') | | | | | | |  |
|  |  | gactTgTtctta | | | | | | |  |
| Hairpin secondary structures at “malignant” mutation sites in Human BRCA1:BRCA1 DNA repair associated | | | | | | | | | |
| BRCA1:BRCA1 DNA repair associated | | NM_007294.4(BRCA1) | | | c.181T>G (p.Cys61Gly) | | | | 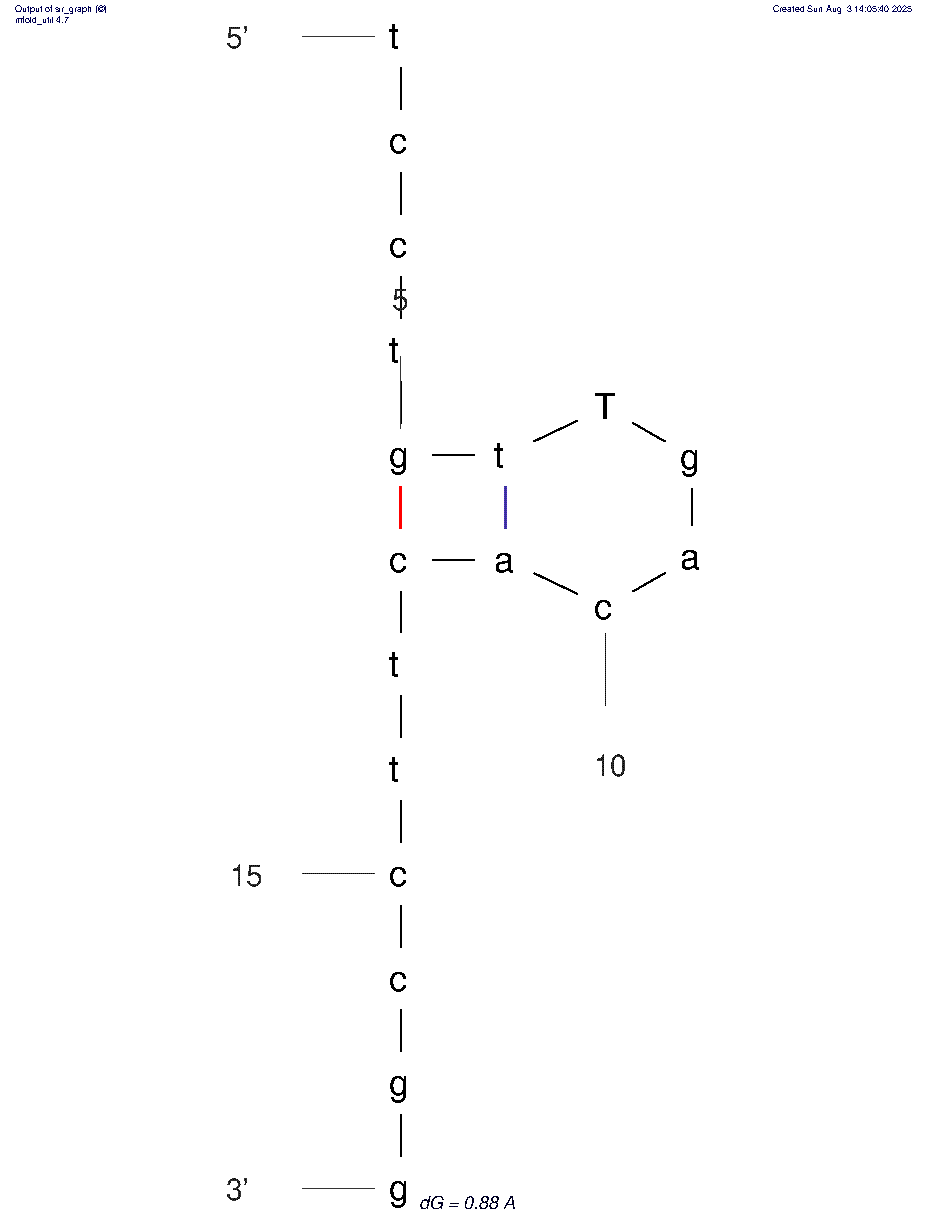  (reverse) |
|  |  | ΔG (kcal/mol) | | ΔH  (kcal/mol) | ΔS (cal/mol·K) | | | Tm (°C) |  |
|  |  | +0.88 | | -19.90 | -67.00 | | | 23.9°C |  |
|  |  | Sequence (5'→3') | | | | | | |  |
|  |  | accagaagaaagggccttcacag[T]tgtcctttatgtaagaa | | | | | | |  |

**Supplementary Table S2 |** Thermodynamic stability of secondary structures formed by oligonucleotides with different stem compositions (stems indicated in brackets), compared with secondary structures formed by CGGC tetramers located in *gyrA* and *katG* at sites of the most frequent resistance-associated mutations

| **Sequence** | **Tetramer** | **ΔG (kcal/mol)** | **ΔH (kcal/mol)** | **ΔS (cal/mol·K)** | **Tm (°C)** |
| --- | --- | --- | --- | --- | --- |
| AA[CGGC]ACCA[GCCG]AA | CGGC  GCCG | –4.33 | –39.60 | –113.72 | 75.1 |
| AA[CGGC]ACCA[GCC]AA | CGGC  GCC | –1.77 | –30.50 | –92.63 | 56.1 |
| AA[CGGG]ACCA[CCCG]AA | CGGG  CCCG | -3.93 | 41.00 | -119.5 | 69.9 |
| AA[GGGC]ACCA[GCCC]AA | GGGC  CCCG | -3.52 | -38.20 | -111.82 | 68.5° |
| AA[CGGC]ACCA[CCG]AA | CGCG  CCG | –2.62 | –29.80 | –87.64 | 66.9 |
| CAA[GGG]ACCA[CCC]AAG  AA[GGG]ACCA[CCC]AA | GGG | -0.81 | –31.60 | -99.27 | 53.6 |
| AA[GGG]ACGCGTACA[CCC]AA (longer loop) | GGG  CCC | –1.11 | –31.60 | –98.31 | 45.2°C |
| AA[GGGG]ACCA[CCCC]AA | GGGG  CCCC | -3.12 | -39.60 | -117.62 | 63.5 |
| CAA[CGG]ACCA[CCG]AAG | CGG  CCG | -2.42 | 33.00 | 98.60 | 61.5 |
| CAA[CGG]ACCA[CCG]AAG | CGG  CCG | -2.01 | -30.20 | -90.89 | 59.1 |
| A[CTTC]ACCA[GAAG]A  (control) | CTTC  GAAG | -1.66 | -35.10 | -107.82 | 52.4 |
| A[CGGC]GACGCG^a^TCGATC  gyrA_p.Ala90Val/Gly^b^ (GCG→GTG)  gyrA_p.Ser91Pro (TCG→CCG)^c^ | CGGC | -3.49 | -30.7 | -91.27 | 63.2 |
| CACCAG[CGGC]^d^  katG p.Ser315Thr (AGC→ACC) | CGGC | 0.21 | -14.5 | -49.34 | 20.7 |
| A[CGGC]GCCGAC  PE_PGRS7 (G302A)^e^ | CGGC | -2.17 | -26 | -79.91 | 52.2 |

**^a^The mutation site is indicated in a box.**

**^b^The *gyrA* p.Ala90Val mutation was detected in 1,468 of 25,838 *Mtb* isolates analyzed^34^.**

**^c^The *gyrA* p.Ser91Pro mutation was detected in 325 of 2,981 *Mtb* isolates analyzed^34^.**

**^d^ The *katG* p.Ser315Thr mutation was detected in 16,552 of 32,154 *Mtb* isolates analyzed^34^.**

^e^ Mutation in PE_PGRS7 in sequenced *Mtb* Beijing B0/W148 (CP049108.1).

**Supplementary Table S3 | Mutator frequencies, CGGC abundance, and distribution of out-of-frame stop codons across bacterial species**

| Species | Frequency of mutators in the population (%) | CGGC abundance,  (%) | Out-of-frame stop codons  (1-nt indels, %) | Out-of-frame stop codons  (2-nt indels,%) |
| --- | --- | --- | --- | --- |
| *Escherichia*  *coli* | 0.5, 1.0, 1.9, 12.1^42^ | 0.55 | 2.08 | 1.67 |
| *Salmonella*  *enterica* | 3.6^42^ | 0.75*^S.typhimurium^*  0.73*^S. typhi^* | 1.9  1.92 | 1.61  1.62 |
| *Pseudomonas*  *aeruginosa* | 19.5, 30, 53.2, 54, 92^42^ | 1.79 | 0.18 | 0.98 |
| *Hemophilus*  *influenzae* | 2.4, 14.5 ^42^ | 0.16 | 2.88 | 2.38 |
| *Neisseria*  *meningitidis* | 22.2, 56.8 ^42^ | 1.11  1.06 | 1.70  1.15 | 1.48  1.44 |
| *Klebsiella*  *pneumoniae* | 14.5 ^42^ | 1.07 | 1.47 | 1.47 |
| *Vibrio*  *parahaemolyticus* | 5.13, 0 ^42^ | 0.27 | 2.33 | 2.07 |
| *Staphylococcus*  *aureus* | 1.4, 4, 14.6 ^42^ | 0.07 | 3.66 | 2.96 |
| *Stenotrophomonas maltophilia* | 21.8 ^42^ | 0.56 | 1.64 | 1.25 |

**Supplementary Table S4 |** List of 88 bacterial genomes, (74 species) included in the study, with corresponding GenBank accession numbers , (NCBI)

|  | Species | Accession code |
| --- | --- | --- |
|  | *Acinetobacter baumannii* | *CP043953.1* |
|  | *Actinomyces israelii strain F0345* | *CP124548.1* |
|  | *Actinomyces naeslundii strain FDAARGOS_1037* | *CP066049.1* |
|  | *Bacillus anthracis str. 'Ames Ancestor'* | *NC007530.2* |
|  | *Bacillus cereus strain FORC_047* | *CP017060.1* |
|  | *Bacteroides fragilis strain FDAARGOS_1225* | *CP069563.1* |
|  | *Bifidobacterium longum subsp. longum JCM 1217* | *NC015067.1* |
|  | *Bordetella parapertussis strain A005* | *CP025070.1* |
|  | *Bordetella pertussis strain H640* | *CP025371.1* |
|  | *Borrelia recurrentis* | *CP000993.1* |
|  | *Borrelia duttonii Ly* | *CP000976.1* |
|  | *Brucella abortus 2308* | *NC007624.1* |
|  | *Burkholderia cepacia strain BC16 chr. 1* | *CP045235.1* |
|  | *Burkholderia cepacia strain BC16 chr. 2* | *CP045236.1* |
|  | *Burkholderia mallei ATCC 23344 chr 2* | *CP000011.2* |
|  | *Burkholderia mallei ATCC 23344 chr. 1* | *CP000010.1* |
|  | *Campylobacter coli str. FDAARGOS_735* | *CP046317.1* |
|  | *Chlamydia pneumoniae Wien1* | *LN846980.1* |
|  | *Chlamydia trachomatis D/UW-3/CX* | *AE001273.1* |
|  | *Citrobacter freundii ATCC 8090* | *CP049015.1* |
|  | *Clostridioides difficile str. S-0253* | *CP076401.1* |
|  | *Clostridium botulinum str. 1169* | *CP013681.1* |
|  | *Corynebacterium diphtheriae str. NCTC11397* | *LN831026.1* |
|  | *Corynebacterium glutamicum ATCC 13032* | *BA000036.3.* |
|  | *Cronobacter sakazakii str. CS-931* | *CP027107.1* |
|  | *Cutibacterium acnes subsp. acnes NBRC 107605* | *AP01623.1* |
|  | *Desulfovibrio desulfuricans str. L4* | *CP072608.1* |
|  | *Eikenella corrodens str. NCTC10596* | *LT906482.1* |
|  | *Escherichia coli O157:H7 str. Sakai DNA* | *NC002695.2* |
|  | *Escherichia coli str. K-12 substr. MG1655* | *NC000913.3* |
|  | *Fusobacterium nucleatum subsp. nucleatum ATCC 25586* | *CP028101.1* |
|  | *Haemophilus influenzae str. FDAARGOS_1560* | *CP085952.1* |
|  | *Klebsiella oxytoca str. NCTC13727* | *LR134333.1* |
|  | *Klebsiella pneumoniae subsp. pneumoniae HS11286* | *CP003200.1* |
|  | *Klebsiella pneumoniae subsp. rhinoscleromatis str. KP4831* | *CP0962.1* |
|  | *Legionella pneumophila str. Philadelphia-1* | *CP013742.1* |
|  | *Leptospira interrogans serovar Copenhageni str. FDAARGOS_203* | *CP020414.2* |
|  | *Listeria monocytogenes EGD-e* | *NC003210.1* |
|  | *Metamycoplasma hominis str. FBG* | *CP055151.1* |
|  | *Moraxella catarrhalis str. CCRI-195ME* | *CP018059.1* |
|  | *Mycobacterium avium subsp. avium str. DSM 44156* | *CP046507.1* |
|  | *Mycobacterium chelonae CCUG 47445* | *CP007220.1* |
|  | *Mycobacterium intracellulare str. FDAARGOS_1564* | *CP085945.1* |
|  | *Mycobacterium kansasii ATCC 12478* | *CP006835.1* |
|  | *Mycobacterium leprae Kyoto-2 DNA* | *AP014567.1* |
|  | *Mycobacterium leprae str. MRHRU-235-G* | *NZ_CP029543.1* |
|  | *Mycobacterium marinum CCUG20998* | *CP024190.1* |
|  | *Mycobacterium tuberculosis str. 11502* | *CP070338.1* |
|  | *Mycobacterium tuberculosis str. 4860* | *CP053092.1* |
|  | *Mycobacterium tuberculosis str. 5005* | *CP049108.1* |
|  | *Mycobacterium tube* *NZ_CP029543.1rculosis str. BLR 4299 2019* | *CP125620.1* |
|  | *Mycobacterium tuberculosis str. BLR-31d* | *CP110674.1* |
|  | *Mycobacterium tuberculosis 9248* | *CP115447.1* |
|  | *Mycobacterium tuberculosis H37Rv* | *NC_000962.3* |
|  | *Mycobacteroides abscessus str. FLAC054* | *CP014961.1* |
|  | *Mycolicibacterium fortuitum subsp. fortuitum JCM6387* | *AP025518.1* |
|  | *Mycoplasma pneumoniae FH* | *CP010546.1* |
|  | *Neisseria meningitidis str. LNP24198* | *CP060280.1* |
|  | *Neisseria meningitidis str. PartJ-Nmeningitidis-RM8376* | *CP064367.1* |
|  | *Nocardia asteroides str. NCTC11293* | *LR134352.1* |
|  | *Porphyromonas gingivalis ATCC 33277* | *NC010729.1* |
|  | *Pseudomonas aeruginosa PAO1* | *NC002516.2* |
|  | *Pseudomonas fluorescens str. NCTC10038* | *LS483372.1* |
|  | *Rickettsia japonica YH* | *AP011533.1* |
|  | *Rickettsia prowazekii str. Breinl* | *CP004889.1* |
|  | *Rhodococcus erythropolis R138* | *CP007255.1* |
|  | *Salmonella enterica subsp. enterica serovar Typhi Ty2* | *AE014613.1* |
|  | *Salmonella enterica subsp. enterica serovar Typhimurium str. LT2* | *NC003197.2* |
|  | *Salmonella Typhimurium str. LT2* | *NC003197.2* |
|  | *Staphylococcus aureus str. BLR-DV* | *CP058312.1* |
|  | *Staphylococcus aureus subsp. aureus* | *NC007795.1* |
|  | *Staphylococcus epidermidis str. ATCC 14990* | *CP035288.1* |
|  | *Staphylococcus haemolyticus str. ATCC 620* | *CP035291.1* |
|  | *Staphylococcus saprophyticus subsp. saprophyticus ATCC 15305* | *NC007350.1* |
|  | *Stenotrophomonas maltophilia NCTC10257* | LT906480.1 |
|  | *Streptococcus agalactiae str. NGBS128* | *CP012480.1* |
|  | *Streptococcus mutans str. FDAARGOS 1458* | *CP077404.1* |
|  | *Streptococcus pneumoniae str. NCTC7465* | *LN831051.1* |
|  | *Streptococcus pyogenes str. NCTC12064* | *LS483338.1* |
|  | *Streptomyces griseus str. NBC_01018* | *CP108682.1* |
|  | *Treponema pallidum subsp. pallidum str. TpN-CL5* | *CP095416.1* |
|  | *Treponema denticola ATCC 35405* | *NC_002967.9* |
|  | *Thermus aquaticus Y51MC23* | *CP010822.1* |
|  | *Veillonella parvula DSM 2008* | *CP001820.1* |
|  | *Vibrio cholerae str. RFB16 chr. 2* | *CP043556.1* |
|  | *Vibrio cholerae str. RFB16 chr. 1* | *CP043554.1* |
|  | *Vibrio parahaemolyticus RIMD 2210633 DNA chr.1* | *BA000031.2* |
|  | *Vibrio parahaemolyticus RIMD 2210633 DNA chr.2* | *BA000032.2* |
